# In-vivo imaging of targeting and modulation of depression-relevant circuitry by transcranial direct current stimulation: a randomized clinical trial

**DOI:** 10.1038/s41398-021-01264-3

**Published:** 2021-02-24

**Authors:** Mayank. S. Jog, Elizabeth Kim, Cole Anderson, Antoni Kubicki, Rishikesh Kayathi, Kay Jann, Lirong Yan, Amber Leaver, Gerhard Hellemann, Marco Iacoboni, Roger P. Woods, Danny J. J. Wang, Katherine L. Narr

**Affiliations:** 1 University of Southern California, Los Angeles, CA USA; 2 University of California, Los Angeles, CA USA; 3 Northwestern University, Chicago, IL USA; 4 University of Alabama at Birmingham, Birmingham, AL USA

**Keywords:** Depression, Neuroscience

## Abstract

Recent clinical trials of transcranial direct current stimulation (tDCS) in depression have shown contrasting results. Consequently, we used in-vivo neuroimaging to confirm targeting and modulation of depression-relevant neural circuitry by tDCS. Depressed participants (*N* = 66, Baseline Hamilton Depression Rating Scale (HDRS) 17-item scores ≥14 and <24) were randomized into Active/Sham and High-definition (HD)/Conventional (Conv) tDCS groups using a double-blind, parallel design, and received tDCS individually targeted at the left dorsolateral prefrontal cortex (DLPFC). In accordance with Ampere’s Law, tDCS currents were hypothesized to induce magnetic fields at the stimulation-target, measured in real-time using dual-echo echo-planar-imaging (DE-EPI) MRI. Additionally, the tDCS treatment trial (consisting of 12 daily 20-min sessions) was hypothesized to induce cerebral blood flow (CBF) changes post-treatment at the DLPFC target and in the reciprocally connected anterior cingulate cortex (ACC), measured using pseudo-continuous arterial spin labeling (pCASL) MRI. Significant tDCS current-induced magnetic fields were observed at the left DLPFC target for both active stimulation montages (Brodmann’s area (BA) 46: *p*_HD_ = 0.048, Cohen’s *d*_HD_ = 0.73; *p*_Conv_ = 0.018, *d*_Conv_ = 0.86; BA 9: *p*_HD_ = 0.011, *d*_HD_ = 0.92; *p*_Conv_ = 0.022, *d*_Conv_ = 0.83). Significant longitudinal CBF increases were observed (a) at the left DLPFC stimulation-target for both active montages (*p*_HD_ = 3.5E−3, *d*_HD_ = 0.98; *p*_Conv_ = 2.8E−3, *d*_Conv_ = 1.08), and (b) at ACC for the HD-montage only (*p*_HD_ = 2.4E−3, *d*_HD_ = 1.06; *p*_Conv_ = 0.075, *d*_Conv_ = 0.64). These results confirm that tDCS-treatment (a) engages the stimulation-target, and (b) modulates depression-relevant neural circuitry in depressed participants, with stronger network-modulations induced by the HD-montage. Although not primary outcomes, active HD-tDCS showed significant improvements of anhedonia relative to sham, though HDRS scores did not differ significantly between montages post-treatment.

## Introduction

Major depression is one of the world’s leading causes of disability. An estimated 20.6% of adults experience a mental illness in the United States each year, with less than a third receiving treatment. Major depression is the most common mental disorder where 8.3% of the adult population has at least one major depressive episode on an annual basis^1^. Though treatable, standard psycho- and pharmacotherapies can be inaccessible or only moderately successful^2–4^. Unfortunately, without treatment or following failed treatment, depressive episodes often become more frequent and last longer^5,6^. Transcranial direct current stimulation (tDCS) is one of an emerging array of neuromodulatory techniques that use mild milliampere currents applied at the scalp to modulate cortical excitability. tDCS is low-cost, non-invasive, induces minimal side effects and has the potential for supervised self-administration^7–9^. Thus if effective, tDCS could be beneficial for lessening the personal suffering and economic burden of major depressive disorder (MDD)^7,10^.

However, recent clinical trials investigating antidepressant effects of tDCS have shown mixed results. In participants with unipolar depression, Brunoni et al. ^11^ showed a significant reduction in mean Hamilton Depression Rating scale (HDRS-17^12^) scores with active anodal tDCS of the left dorsolateral prefrontal cortex (DLPFC) compared to sham at long-term follow-up. However, Loo et al. ^13^ observed more remitters in the sham group compared to the active left DLPFC tDCS treatment group. A diagnosis of depression can encompass a diverse combination of symptoms across individuals^14^. However, while contrasting findings could be due to the heterogeneity of depression, mixed results may also be attributable to differences in the tDCS montage, anatomical accuracy of targeting, and other stimulation parameters^15^. For example, in the two powered clinical trials cited above with contrasting results^11,13^, there were differences in electrode placement (OLE F3/F4 vs. F3/F8), electrode size (5 × 5 vs. 5 × 7 cm) and dose (2 vs. 2.5 mA). These and other disparate findings concerning the efficacy of tDCS^10^ highlight the need to confirm the (a) in-vivo targeting of brain tissue by tDCS, defined as validating the delivery of tDCS at the brain target and the (b) long-term modulation of neural circuitry, defined here as tDCS-induced neurobiological changes following a course of tDCS-treatment. Addressing whether a particular tDCS montage can target and modulate depression-relevant circuitry is paramount to optimizing future tDCS treatment protocols. This way, even if subsequent trials show no systematic therapeutic effects, the confirmed engagement of neural circuitry can rule out the role of treatment montages. These outcomes may then motivate research into interventions targeting precise behavior-related dysfunctional circuits. To the best of our knowledge^16^ no study has yet demonstrated whether depression-relevant prefrontal-limbic circuitry, including the DLFPC and the reciprocally connected anterior cingulate cortex (ACC)^17,18^, is targeted and modulated post left DLPFC tDCS-treatment in patients with major depression.

The present study was designed to answer the question of tDCS target engagement and modulation by evaluating the cerebral effects of two tDCS montages (a high-definition “HD” montage^19,20^, and a “Conventional” montage^21,22^). Here, the anode was centered at the left DLFPC, the same brain region targeted by the majority of previous depression studies (i.e. refs. ^11,13,23–27^), using stereotaxic mapping of brain coordinates in each individual. We hypothesized that Active-tDCS targeted at the left DLPFC would induce significant magnetic fields during stimulation in the same region, according to Ampere’s Law (hypothesis#1). Previous research suggests DLPFC tDCS affects behaviors involving brain networks implicated in depression. For example, left DLPFC stimulation has been shown to affect executive function, cognitive control, and response inhibition involving prefrontal association areas, including the reciprocally connected ACC that integrates information from subcortical limbic structures^28–30^. Further, DLPFC tDCS can influence downstream emotional processing^31–33^ including the appraisal of emotionally valenced stimuli^34,35^ and negativity bias^36^. Notably, existing data also suggest that the DLPFC and ACC are modulated by other antidepressant treatments^37–42^. Consequently, we hypothesized that anodal left DLFPC tDCS may lead to the modulation of DLPFC and ACC neural properties measurable as changes in CBF (hypothesis#2). To complement our primary outcome measures, an exploratory analysis evaluated neural engagement during stimulation by investigating the concurrently acquired tDCS-induced blood-oxygenation-level-dependent (BOLD) response. Other exploratory analyses investigated the tDCS effects on depressive symptoms and anhedonia, and whether baseline neural engagement could serve as a potential biomarker for treatment response.

## Methods

**Fig. 1:**
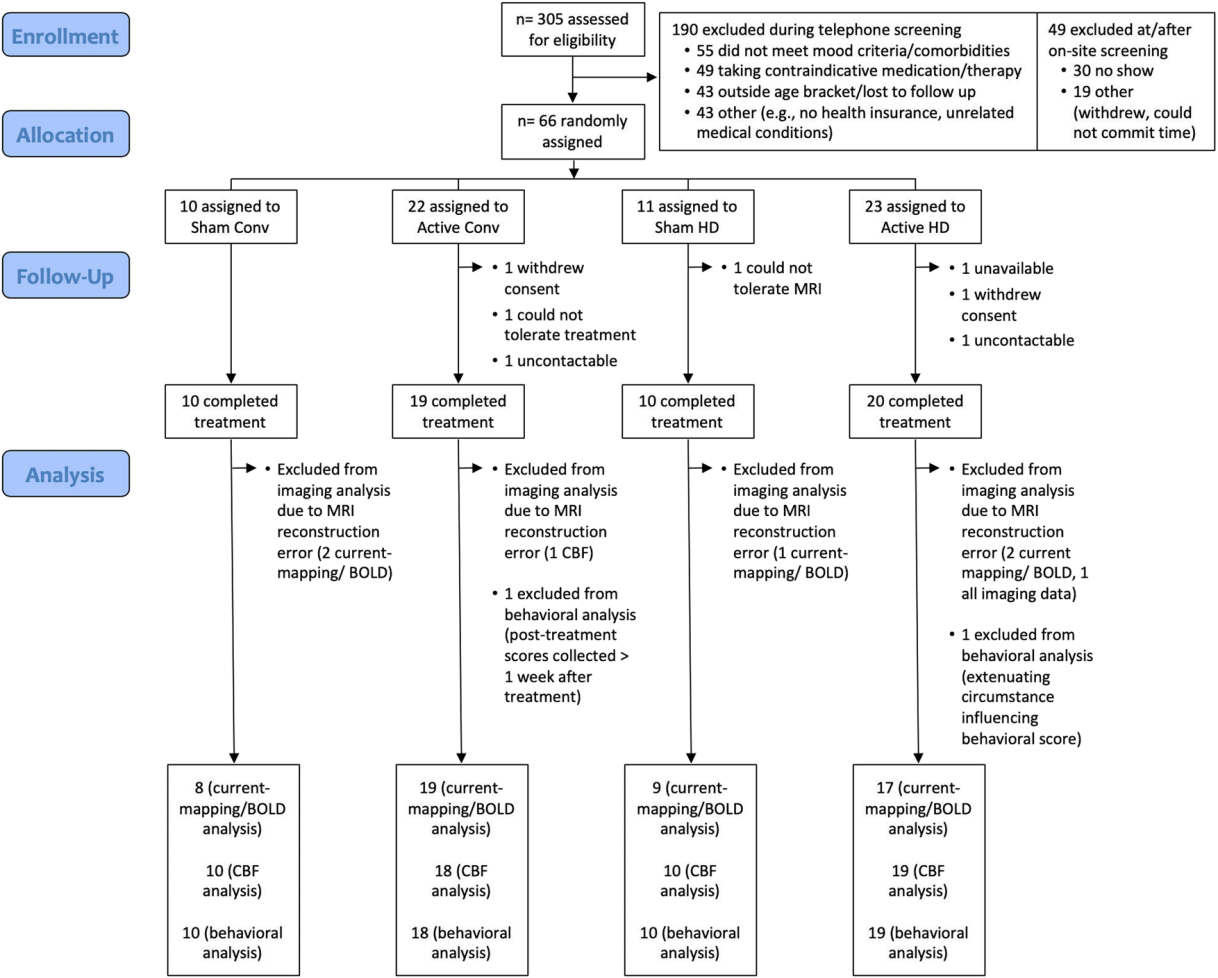
**CONSORT diagram.** Three hundred and five participants were assessed for eligibility, of which 239 participants were excluded at screening (for a full list of exclusionary criteria, see supplementary material S1). Post screening, 66 participants were enrolled and randomly assigned to treatment groups (Sham Conventional (Conv), Active Conv, Sham high-definition (HD), and Active HD tDCS). Data was analyzed for the participants that completed treatment (*N* = 59). Out of these, imaging data from six current-mapping/BOLD datasets and two pCASL CBF datasets was excluded due to MRI-reconstruction errors. Additionally, behavioral data from two participants was excluded because of extenuating circumstances that delayed the collection of scores and the occurrence of a family-emergency, respectively.

This tDCS clinical trial (NCT03556124^43^) was conducted at the University of California, Los Angeles (UCLA) from October 1, 2017 to December 31, 2019. As shown in Fig. 1, a parallel design was used, and participants successfully screened for inclusion/exclusion criteria were randomly assigned to Active/Sham X HD/Conventional tDCS conditions using a computer-generated list while stratifying for sex.

### Participants

A total of 305 participants from the Los Angeles area were assessed for eligibility. Included participants were required to meet criteria for a current major depressive episode confirmed using the Structured Clinical Interview for DSM-5^44^, and were required to (a) be between 18 and 55 years old, (b) have a HDRS score of ≥14 and <24, and (c) be treatment naïve, or on a stable standard antidepressant regimen (including selective serotonin reuptake inhibitors (SSRIs), serotonin-noradrenaline reuptake inhibitors (SNRIs), monoamine oxidase inhibitors (MAOIs) or tricyclics (TCAs)) with no change in treatment 6-weeks prior to and during the tDCS intervention. Participants with severe or treatment-resistant depression (HAMD scores ≥24 and a history of a major depressive episode lasting >2-years or failure to 2 or more antidepressant trials in the current index episode) were excluded. More detailed inclusion and exclusion criteria are described in Supplementary material S1.

Of the 66 participants enrolled in the study, 59 completed treatment and all research measures (Fig. 1) with *n* = 20 participants in Active HD condition, *n* = 19 in Active Conventional condition, and *n* = 20 in the Sham group (*n* = 10 each for HD Sham and Conventional Sham montages, respectively). Of the acquired brain scans, six current-mapping/BOLD datasets and two CBF datasets were excluded because of MRI reconstruction errors. Finally, clinical/behavioral data from two participants were excluded due to participant unavailability or family bereavement. The remaining participants were all included in the intention-to-treat analysis. The clinical/demographic characteristics of participants are shown in Supplementary Table S2. Informed consent was obtained from all participants following approval of study procedures by the UCLA Institutional Review Board (IRB).

### tDCS treatment

**Fig. 2:**
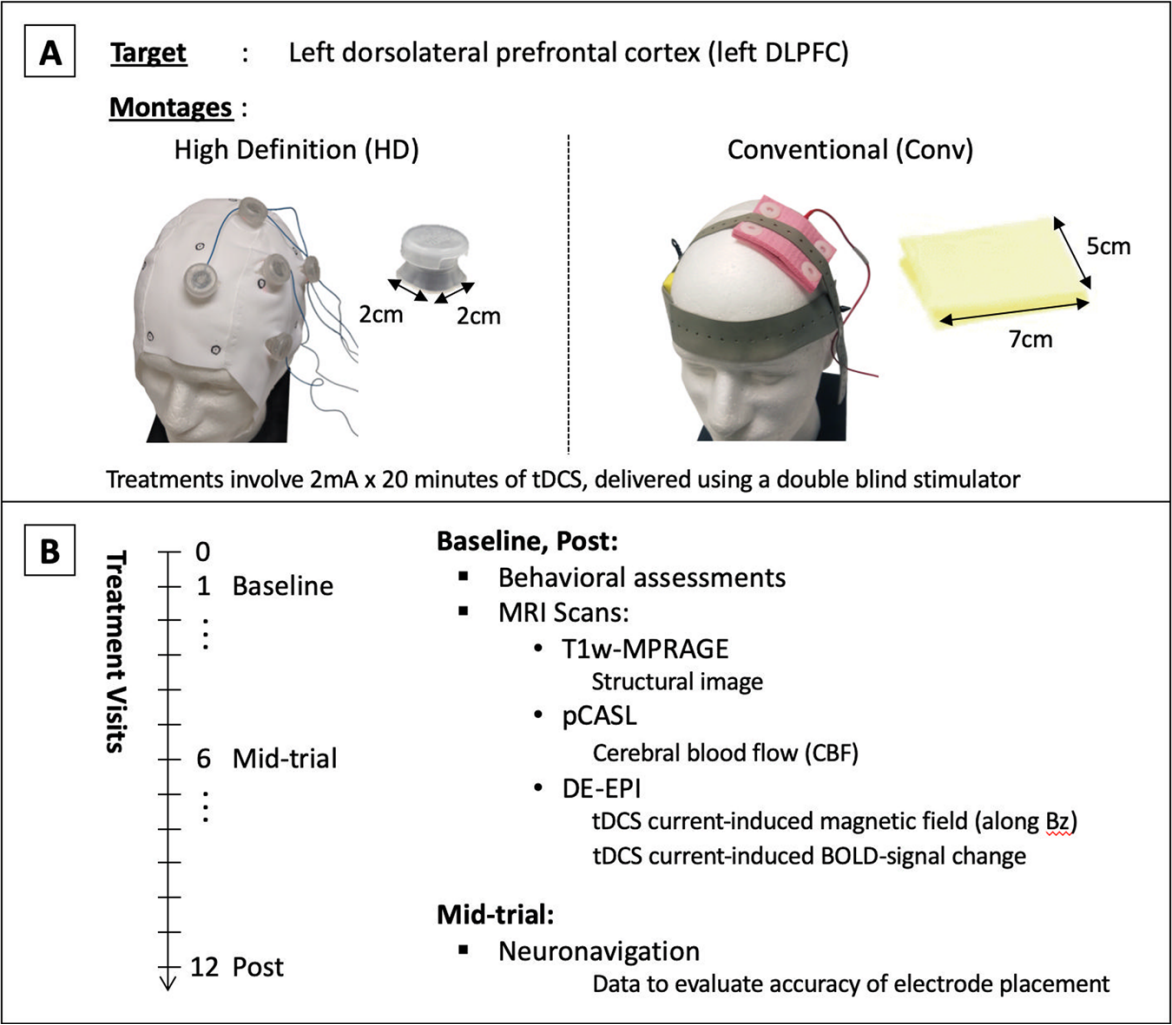
**tDCS stimulation parameters and data acquisition protocol.** **A** Shows the high-definition (HD) and conventional (Conv) tDCS montages utilized in the study. The HD montage consists of a central anode electrode delivering 2 mA at the stimulation target, with four return electrodes placed 5 cm away and equidistant from each other. The Conv montage consists of a 7 × 5 cm electrode placed on the stimulation target, with the return electrode placed at [*x* = 56, *y* = 30, *z* = −1] MNI coordinates (~F8). The stimulation target was selected to be [*x* = −46, *y* = 44, *z* = 38] MNI coordinates, and electrode placement was performed as detailed in Supplementary material S3. **B** Shows the data acquisition protocol over treatment visits for each participant. Visit 0 was the consult visit, where a T1w-MPRAGE MRI was acquired to guide electrode placement. Behavioral and MR-imaging data used in analysis were acquired at visits 1 and 12 (baseline and post-treatment, respectively). MRI data included a second structural T1w-MPRAGE scan (used in simulations), followed by a pseudo-continuous arterial spin labeling scan (pCASL, used to calculate cerebral blood flow (CBF)), and a dual-echo echo planar imaging scan (DE-EPI, used to calculate (a) the tDCS current-induced magnetic field along the MRI static field (Bz) and (b) the tDCS current-induced BOLD-response). TDCS was delivered during the DE-EPI scan only, which was performed after the pCASL scan to preclude any acute CBF changes resulting from tDCS stimulation. Neuronavigation data was also acquired using Brainsight at the mid-trial visit (visit 6) to quantify the accuracy of electrode-positioning relative to the stimulation target.

Treatments were designed to deliver tDCS individually targeted at the left DLPFC in each participant. Specifically, the Brainsight neuronavigation system^45^ was used to select the stimulation-target as the scalp projection of [*x* = −46, *y* = 44, *z* = 38] MNI coordinates^46,47^ based on structural MRIs acquired from each participant. Each participant received tDCS from one of two montages: (a) the HD montage, or the (b) Conventional (Conv) montage, as illustrated in Fig. 2A. For both montages, the anode electrode was centered over the stimulation-target.

A common 4 × 1 ring arrangement was used for the HD-montage, with the anode at the stimulation-target and the four cathodes placed 5 cm away and equidistant from each other^19,20^. For the Conventional montage, a bicephalic montage with 7 × 5 cm electrodes was used, with the anode positioned over the stimulation-target and the cathode placed over the scalp projection of [*x* = 56, *y* = 30, *z* = −1] MNI coordinates (~F8). A controlled electrode placement procedure was employed (described in Supplementary material S3), and placement accuracy was evaluated at visit 6 using Brainsight software^45^.

For randomization, each participant was assigned a code at enrollment, and these codes were used to operate a double-blind stimulator (Soterix, Model#5100D) to administer tDCS. Both participants and assessors were blind to the stimulation condition (Active/Sham) and were polled after each participant’s final treatment-session to verify the integrity of blinding. It was, however, not possible to institute blinding of montage-type (HD or Conv). Participants received tDCS for 12 consecutive working days similar to previous studies in depression^48–51^. Active-tDCS involved 2 mA × 20 min of stimulation^10^. Sham-tDCS involved a ramp up to 2 mA followed immediately by a ramp-down at the beginning of each treatment-session. Apart from these brief periods of ramping, ammeter readings showed that the device emitted a steady current of 65 μA during sham-tDCS; note that this current is an order of magnitude smaller than active-stimulation, and is similar to the sham protocol of Loo et al. ^13^. All ramp-times were 30 s.

### Data acquisition and preprocessing

Figure 2B shows the schedule of data acquisition alongside the treatment visits. At visit 0, a T1-MPRAGE structural MRI was acquired to guide electrode placement. At visits 1 and 12 (baseline and post-treatment), data from a T1-MPRAGE, 3D GRASE pseudo-continuous arterial spin labeling (pCASL) scan^52^, and a dual-echo echo planar imaging (DE-EPI) scan^53^ were acquired. Note that tDCS was applied in real-time during the DE-EPI scan. Thus, DE-EPI was performed after the pCASL scan to preclude any acute CBF changes resulting from tDCS currents. The full sequence parameters and tDCS currents employed in DE-EPI are described in Supplementary Fig. S4.

Although this study was not designed or powered to address clinical efficacy, the HDRS-17^12^ and the Snaith Hamilton Pleasure scale (SHAPS^54^) were also acquired at baseline and post-treatment. The HDRS provided a measure of overall symptom severity, while the SHAPS measured anhedonia, a core symptom of depression. Finally, Brainsight^45^ was used to measure the electrode position relative to the stimulation-target at visit 6 to quantify positioning accuracy.

MRI data was preprocessed using established methods as described in Supplementary material S5. Briefly, all data was corrected for motion using SPM12 realignment procedures. Magnetic field data from the DE-EPI scan was modeled using a general linear model (GLM) with the applied current as a predictor. The BOLD data from DE-EPI was modeled similarly with the applied current convolved with the hemodynamic response function as a predictor. These predictor estimates can be interpreted as the tDCS current-induced magnetic fields and BOLD-signal changes, respectively (in nT/mA tDCS, and a.u., respectively)^53^. The pCASL data was modeled using the general kinetic model for perfusion^55^ to quantify CBF (in ml/100 g/min). These maps were coregistered to the structural MRI of the same participant and normalized to the MNI space for group-level statistics using SPM12.

### Statistical analysis

The sample size was estimated to achieve 80% power with alpha <0.05 (two-tailed) for an effect size of *d* = 0.91 when comparing groups pairwise (*n* = 20 each), or *d* = 0.78 when comparing Active-stimulation and Sham groups (*n* = 40 and *n* = 20, respectively). Between-group differences in clinical/demographic characteristics and electrode-placement were tested using one-way ANOVA and *χ*^2^-tests (for continuous and categorical random variables respectively). A *χ*^2^-test was also used to confirm the integrity of blinding by testing differences in guesses of Active/Sham stimulation across allocation groups in participants and assessors.

TDCS current-induced magnetic fields were measured in two regions-of-interest (ROIs) encompassing the left DLPFC target (Brodmann areas (BA) 46 and 9 defined using the Sallet atlas^56^). Next, stray fields, estimated using the Biot–Savart law applied on the current-carrying wires (described in Supplementary material S6), were subtracted from the ROI-averaged fields as recommended in previous studies^57,58^. Finally, the stray field-corrected ROI-averaged fields were averaged over baseline and post-treatment DE-EPI scans in the same participant and compared using a one-way ANOVA between Active-HD, Active-Conv, and Sham treatment groups.

In addition to investigating current-induced magnetic fields, an exploratory analysis was performed to determine concurrent changes in BOLD activity during tDCS. Here, the tDCS-induced BOLD-signal change was modeled voxel-wise at the group-level using a full factorial model (SPM12), and a t-contrast was used to investigate changes between the Active (HD+Conv) and Sham groups. Only baseline visit data was included in this analysis to preclude time/tDCS-treatment effects.

Next, to determine longitudinal changes in targeted neural circuitry, CBF-maps were modeled voxelwise in a full factorial model using SPM12, and a t-contrast was used to investigate changes in CBF (post-pre treatment) between Active (HD+Conv) and Sham groups. Montage-specific changes were investigated by comparing Active-HD and Active-Conv groups in a t-contrast. Differences in baseline CBF were similarly tested between the same groups. Since the DE-EPI scan with concurrent tDCS stimulation occurred after the CBF measurements (Fig. 2B), changes in CBF calculated as the difference between baseline and post-treatment CBF measurements reflect neuroplastic changes after participants have received 11 tDCS treatments. Consequently, the CBF changes measured in this study capture changes over the course of all tDCS treatment sessions and are referred to as persistent post-treatment CBF changes hereon.

Finally, we explored whether %changes in HDRS and SHAPS (calculated as (post-pre)/pre*100, and referred to as %ch-HDRS and %ch-SHAPS hereon) over the course of the trial differed between Active-stimulation and Sham groups using 2-sample *t*-tests. If both clinical scores and tDCS-induced BOLD-signal near the stimulation-target changed significantly relative to sham, correlations between these measures were investigated in post-hoc analyses to identify potential biomarkers for future research.

For the ROI analysis investigating tDCS current-induced magnetic fields at the left DLPFC stimulation target (Hypothesis #1), Bonferroni correction at *p* < 0.05 was used to correct for multiple comparisons. For Hypothesis #2 investigating whether tDCS treatment induces longitudinal CBF changes in the left DLPFC stimulation target and the reciprocally connected ACC, the voxel-wise analysis was thresholded at *p* < 0.01, cluster-size >50 and corrected for multiple comparisons using small volume correction (SVC^59^) at *p* < 0.05. The SVC was performed using anatomical ROIs from the Sallet^56^ and Freesurfer–Destrieux^60^ atlases. A complementary analysis using the average CBF values in the same anatomical-ROIs was also performed to support the voxel-wise results (shown in Supplementary Fig. S7). The voxel-wise analysis helps in localizing the peak CBF changes for comparison with the coordinates of the stimulation-target. Remaining exploratory analyses investigating clinical scores were thresholded at *p* < 0.05, and the voxelwise tDCS-induced BOLD signal results were thresholded at *p* < 0.01, cluster-size >50 and limited to the left DLPFC (as identified by anatomical ROI from the Sallet atlas^56^) to complement and augment the primary measures used to investigate targeting (magnetic fields) and modulation (change in CBF) of the left DLPFC stimulation-target.

## Results

No significant differences in age, sex, and other clinical and demographic characteristics were observed between the Active-HD, Active-Conv, and Sham groups (Supplementary Table S2). Similarly, no significant differences between the same groups were observed for (a) electrode-displacements from stimulation-target (*p* = 0.81; mean = 7.0 mm, SD = 2.8 mm, max = 13 mm overall), or (b) guesses of Active/Sham stimulation for participants and assessors (Participants: *χ*^2^ = 1.54, *p* = 0.46; Assessors: *χ*^2^ = 0.045, *p* = 0.97) (Supplementary Table S2).

**Fig. 3:**
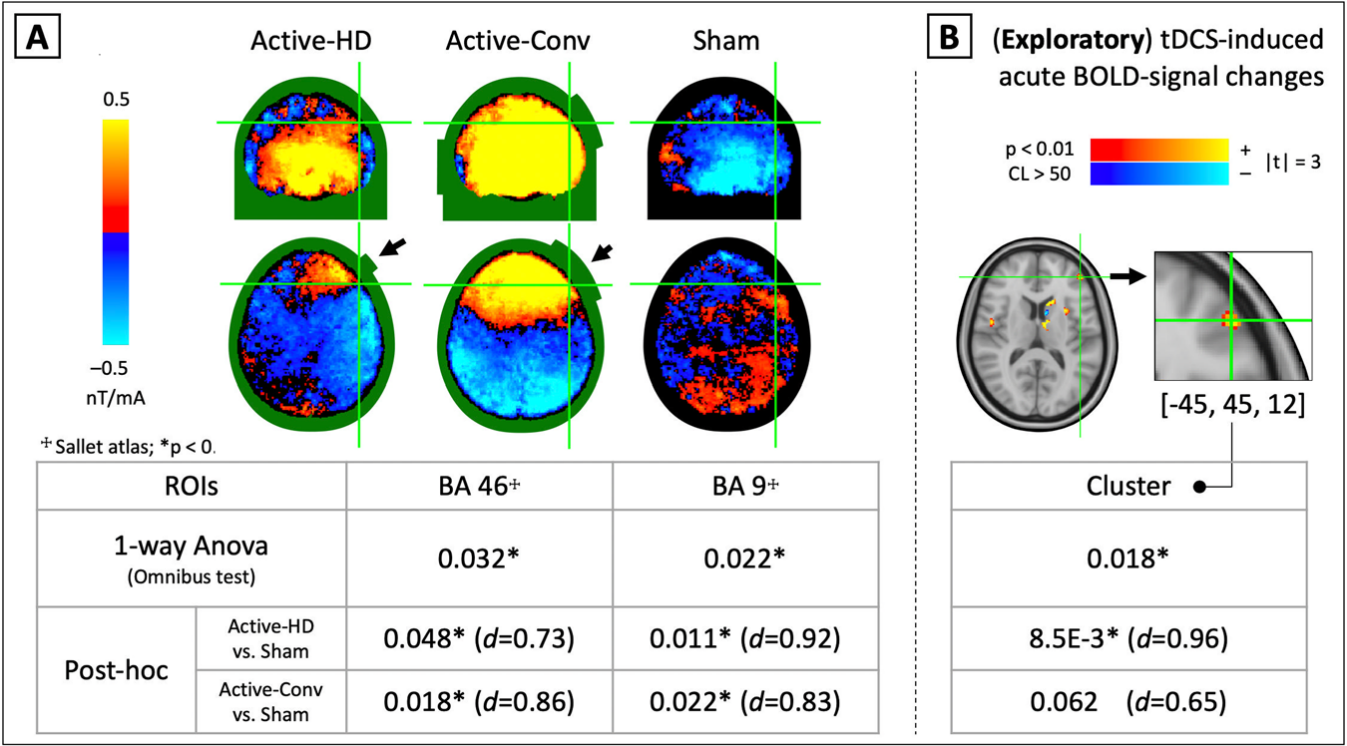
**In-vivo evidence of tDCS-delivery on target.** **A** shows the average tDCS current-induced magnetic fields in the Active-HD, Active-Conv, and Sham groups, with the electrode positions indicated by black arrows. The systematic sign-boundary near the electrodes indicates the “center-of-mass” of current, according to Fleming’s right-hand rule. Quantitatively, the average field in two regions of interest encompassing the left DLPFC (Brodmann areas (BAs) 46 and 9, selected from the Sallet atlas^56^) were assessed across groups in a one-way ANOVA. Corrected for multiple comparisons using the Bonferroni criterion, both regions show significant current-induced magnetic field differences between conditions, with posthoc *t*-tests revealing significantly larger fields for both Active-stimulation montages compared to Sham with medium-to-large effect sizes (Posthoc *t*-tests: BA 46: *d*_HD_ = 0.73, *d*_Conv_ = 0.86; BA 9: *d*_HD_ = 0.92, *d*_Conv_ = 0.83; *p* < 0.05 in all cases). **B** While significant tDCS current-induced magnetic fields in the left DLPFC indicate delivery of tDCS on target, tDCS-induced BOLD-signal changes in the same region could indicate neural-engagement during stimulation. An exploratory analysis investigating tDCS-induced BOLD signal changes between Active (HD+Conv) and Sham groups was performed, and significant clusters (Active > Sham) largely lateralized to the stimulated left-hemisphere were observed (*p* < 0.01, cluster-size > 50). In particular, a significant cluster was observed at [*x* = −45, *y* = 45, *z* = 12] (peak-voxel) in BA 46, a region confirmed to be targeted from magnetic field measurements (**A**). Here, posthoc *t*-tests showed a significant effect for Active-HD with a large effect size, and a trending medium-to-large effect-size for the Active-Conv group compared to Sham ([HD]: *d* = 0.96, *p* = 8.5E−3, [Conv]: *d* = 0.65, *p* = 0.062), indicating a systematic neurophysiological response at the stimulation target with the applied current. Note that the current-induced magnetic fields used in the analysis were corrected for stray-fields following the approach of Goksu et al. ^57,58^ (described in Supplementary material S6). A confound-analysis was also performed for the BOLD data (following the approach of Jog et al. ^53^, shown in Supplementary Fig. S12), and indicated that the measured tDCS-induced BOLD-signal change was likely neurophysiological in origin.

Figure 3A shows the average tDCS current-induced magnetic fields for the Active-HD, Active-Conv, and Sham groups. The sign boundary near the anode electrodes in Active-HD and Active-Conv (marked with arrows) shows the center-of-mass of current (intuited by Fleming’s right-hand rule^61^) over the left DLPFC stimulation-target. Quantitatively, the average magnetic fields in BA 46 and 9 were observed to differ significantly between groups (BA 46 *p* = 0.032; BA 9 *p* = 0.022), with posthoc *t*-tests showing significant fields for both Active-stimulation montages with a medium-to-large effect-size (BA 46 [HD]:confidence interval (CI) = (5.8E−3,1.6), *d* = 0.73, *p* = 0.048; [Conv]:CI = (0.19,1.9), *d* = 0.86, *p* = 0.018), and BA 9 [HD]:CI = (0.22,1.6), *d* = 0.92, *p* = 0.011; [Conv]:CI = (0.16,1.9), *d* = 0.83, *p* = 0.022).

Figure 3B shows the exploratory analysis investigating tDCS-induced BOLD-signal changes in the left DLPFC region. Peak-voxel for this significant cluster was observed to be at [*x* = −45, *y* = 45, *z* = 12] in BA 46 near the stimulation-target. Here, posthoc *t*-tests showed a significant effect for Active-HD versus Sham with a large effect-size, and a medium-to-large effect-size for Active-Conv versus Sham at trend-level significance ([HD]:CI = (0.22,1.4), *d* = 0.96, *p* = 8.5E−3; [Conv]:CI = (−0.032,1.2), *d* = 0.65, *p* = 0.062). MNI coordinates for all significant clusters (including the left DLPFC) are reported in Supplementary Material S8 (see figure and table).

**Fig. 4:**
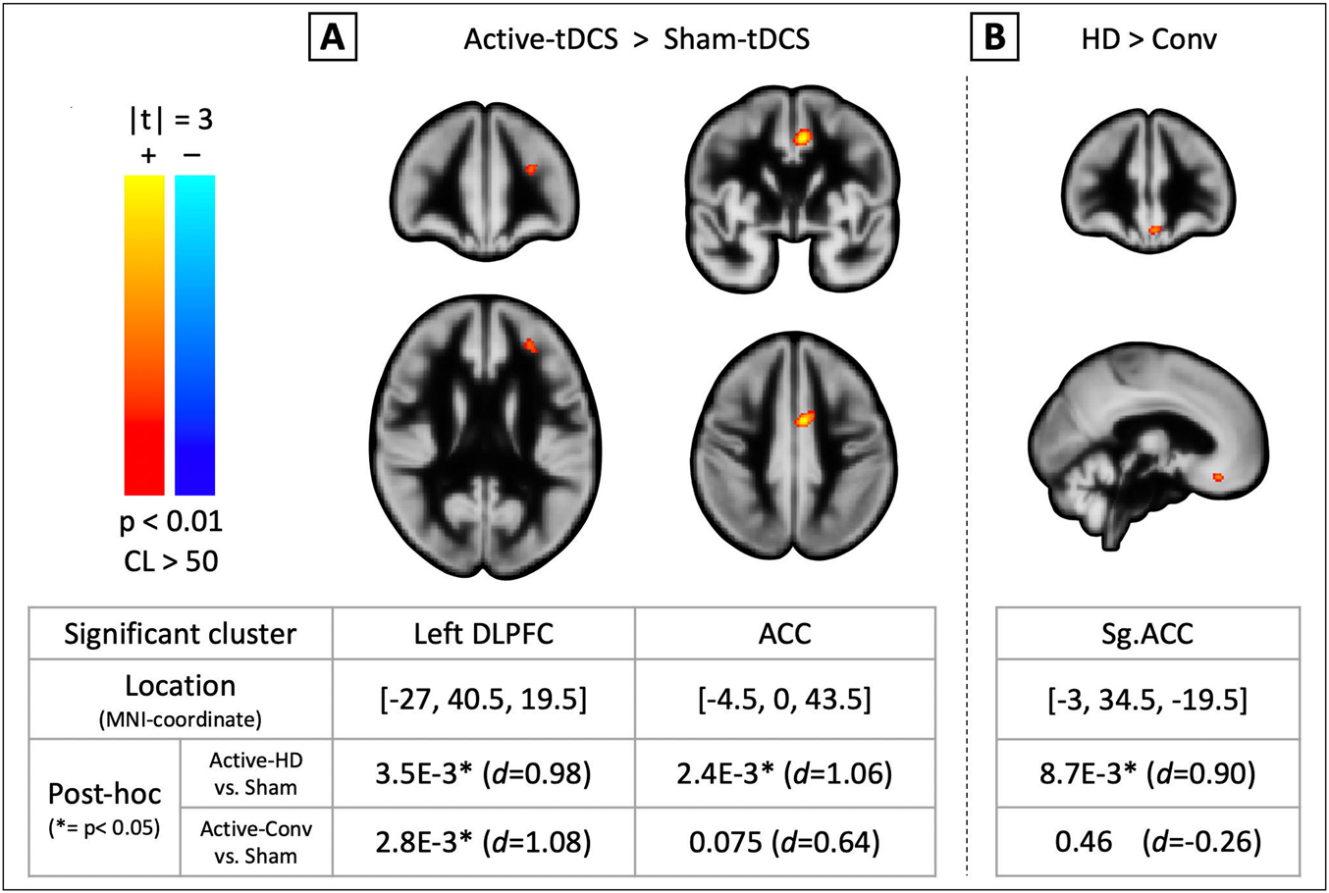
**tDCS treatment-induced changes in cerebral blood flow.** **A** shows post-treatment CBF changes in the Active-stimulation group relative to Sham over the brain. Two significant clusters were observed (*p* < 0.01, cluster-size > 50). The first, at [*x* = −27, *y* = 40.5, *z* = 19.5] MNI coordinates, when compared to the stimulation target at [*x* = −46, *y* = 44, *z* = 38], is (a) 3.5 mm distance away in the coronal direction (i.e. <1/2FWHM used for spatial-smoothing), and (b) 44.2° in the coronal plane under the target; indicating that the observed cluster is located directly underneath the stimulation-target. Here, posthoc *t*-tests show that both montages induce a significant increase in CBF (relative to Sham) with large effect-sizes (*d*_Conv_ = 1.08, *d*_HD_ = 0.98; *p* < 0.05 for both). The second cluster is located at [*x* = −4.5, *y* = 0, *z* = 43.5] MNI coordinates. Here, posthoc *t*-tests showed a significant effect for Active-HD with a large effect size, and a trending medium-to-large effect-size for Active-Conv compared to Sham ([HD]: *d* = 1.06, *p* = 2.4E−3; [Conv]: *d* = 0.64, *p* = 0.075). Overall, these clusters are part of BA 46 in the left DLPFC and the ACC, respectively, which in turn are part of the prefrontal-limbic network (see Supplementary Fig. S7 for complementary anatomical-ROI analysis showing similar results). **B** shows montage-specific CBF changes over the brain (Active-HD > Active-Conv). Here, a significant cluster was observed at [*x* = −3, *y* = 34.5, *z* = −19.5] MNI coordinates in the subgenual-ACC. Follow-up posthoc *t*-tests showed a significant effect for Active-HD with a large effect size, and non-significant results for Active-Conv relative to Sham ([HD]:*d* = 0.90, *p* = 8.7E−3; [Conv]: *d* = −0.26, *p* = 0.46). Note that no significant clusters indicating differences in baseline-CBF were observed between the compared groups.

Figure 4A shows post-treatment CBF changes in the Active-stimulation groups (Conv and HD) compared to Sham (Active > Sham). Here, two significant clusters were observed at [*x* = −27, y = 40.5, *z* = 19.5] and [*x* = −4.5, *y* = 0, *z* = 43.5] (peak-voxels) in BA 46 and the ACC, respectively (according to the Sallet^56^ and Freesurfer–Destrieux^60^ atlases). Posthoc *t*-tests for the first cluster showed significant CBF increases post-treatment for both active-stimulation montages with large effect-sizes ([HD]:CI = (2.7,12.9), *d* = 0.98, *p* = 3.5E−3; [Conv]:CI = (3.2,14.3), *d* = 1.08, *p* = 2.8E−3). For the second cluster, posthoc *t*-tests showed a significant effect for Active-HD with a large effect-size, and a trending medium-to-large effect-size for Active-Conv compared to Sham ([HD]:CI = (3.6,15.5), *d* = 1.06, *p* = 2.4E−3; [Conv]:CI = (−0.58,11.5), *d* = 0.64, *p* = 0.075).

Figure 4B shows montage-specific post-treatment changes in CBF (Active-HD > Active-Conv). A single significant cluster was observed at [*x* = −3, *y* = 34.5, *z* = −19.5] (peak-voxel) in the subgenual ACC (sgACC). Here, posthoc *t*-tests showed a significant increase in CBF for Active-HD with a large effect-size, and non-significant effects for Active-Conv compared to Sham ([HD]:CI = (2.09,13.5), *d* = 0.90, *p* = 8.7E−3; [Conv]:CI = (−7.69,3.58), *d* = −0.26, *p* = 0.46). Finally, no significant clusters indicating differences in baseline CBF were observed in any of the groups compared above.

Although both HD and conventional montages induced similar CBF increases at the left DLPFC stimulation-target, a differential response to stimulation was observed in distal regions. To rule out participant discomfort as a potential cause of these differences, we performed post-hoc analyses to test differences in stimulation-related participant discomfort using a modified Generic Assessment of Side Effects (GASE) scale^62^ that included the following items: headache, dizziness, palpitations, breathing difficulty, nausea, rash, fever, and fatigue. No significant differences between sham, active-conventional and active-HD groups were observed (*p* = 0.20, see Supplementary Fig. S9 for details).

**Fig. 5:**
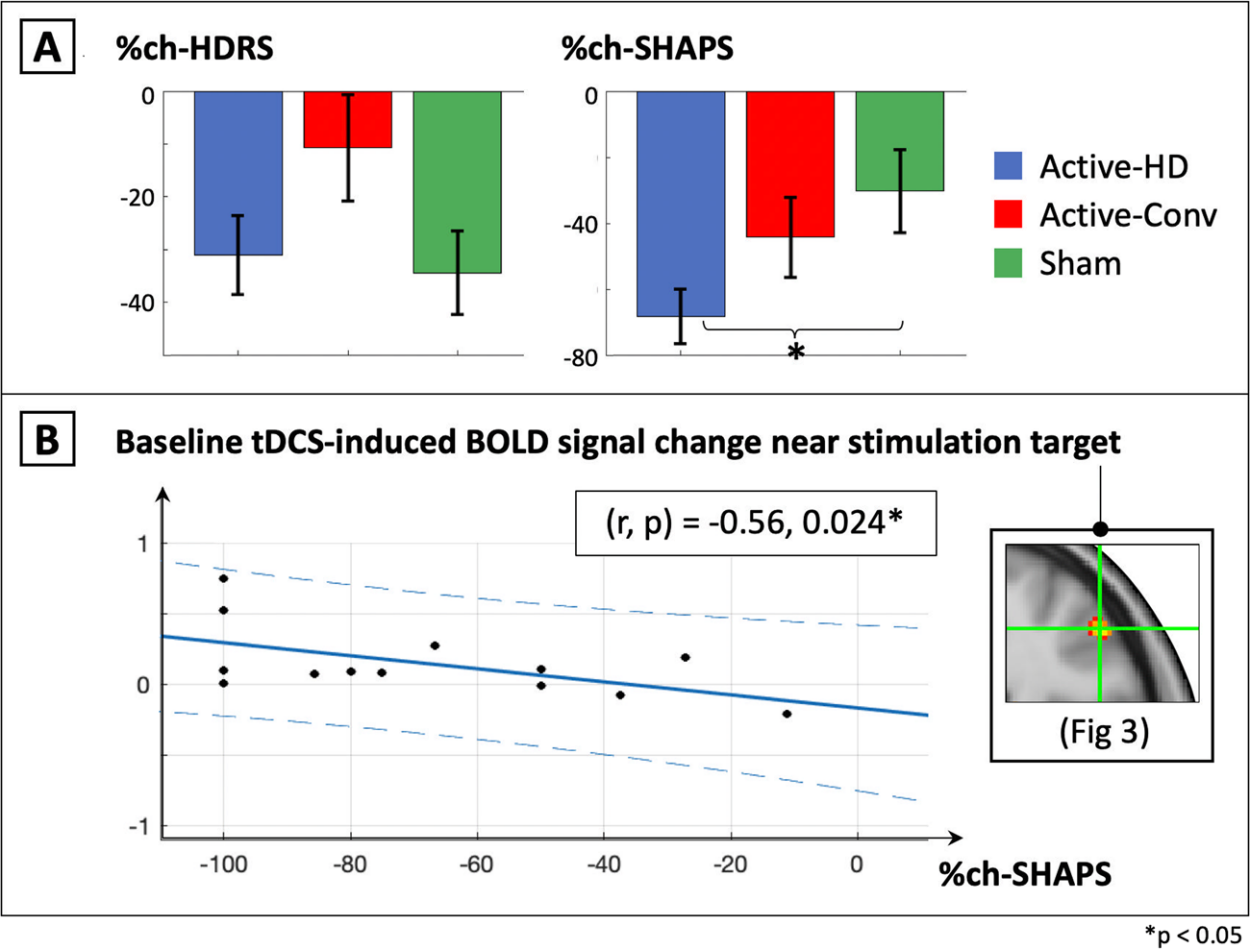
**Mood scores.** **A** shows no significant differences observed for %change in HDRS between Active-HD, Active-Conv, and Sham groups ([HD]: *d* = 0.11, *p* = 0.76; [Conv]: *d* = 0.62, *p* = 0.08). Significant differences between Active-HD and Sham were observed for the Snaith Hamilton Pleasure Scale (SHAPS) measuring anhedonia, a core feature of depression ([HD]: *d* = −0.85, *p* = 0.019; [Conv]: *d* = −0.28, *p* = 0.43). Also for the HD-group, a significant correlation was observed between %ch-SHAPS and the tDCS-induced BOLD-signal change near the stimulation target (shown in **B**, *r* = −0.56, *p* = 0.024). Note that the negative sign of the correlation indicates that the greater the tDCS-induced BOLD-signal change near the stimulation target at baseline, the larger the reduction in SHAPS, indicating improvement. Finally, no significant correlations were observed between %ch-SHAPS and the measured tDCS-current induced magnetic field or the simulated current-density from the same region (shown in Supplementary Fig. S13).

Figure 5A shows results from the exploratory analysis comparing %ch-HDRS and %ch-SHAPS between Active-stimulation groups and Sham. For %ch-HDRS, non-significant and trending effects were observed for Active-HD and Active-Conv (respectively) compared to Sham ([HD]:CI = (−18.8,25.7), *d* = 0.11, *p* = 0.76; [Conv]:CI = (−2.92,50.3), *d* = 0.62, *p* = 0.08). The response rate was 30%, 26.3% and 40% in the sham, active-conv and active-HD groups respectively, and the remission rate was 25%, 10.5%, and 25% for the same (responders defined as %participants whose HAMD scores improved by >50%, and remitters defined as %participants whose post-treatment HAMD was ≤ 7) (Supplementary Fig. S10). For %ch-SHAPS, the Active-HD group showed significant improvements in anhedonia compared to Sham with a large effect size, while non-significant effects were observed between Active-Conv and Sham ([HD]:CI = (−69.7,−6.6), *d* = −0.85, *p* = 0.019; [Conv]:(CI = (−50.1,21.8), *d* = −0.28, *p* = 0.43). Finally, a significant negative correlation was observed in the Active-HD group between %ch-SHAPS and the tDCS-induced BOLD response at baseline near the stimulation-target (Fig. 5B; *r* = −0.56, *p* = 0.024).

## Discussion

In this randomized, double-blinded clinical trial, neuroimaging data was collected pre and post 12 tDCS treatment sessions in participants with depression, and the in-vivo targeting of tDCS currents and treatment-induced changes in CBF were evaluated. Participants were randomized to receive HD or conventional tDCS with the anode individually positioned to target the left DLPFC using neuronavigation and rigorous electrode placement procedures. We focused on the DLPFC because altered prefrontal-subcortical circuitry is widely implicated as contributing to the pathophysiology of depression^63–65^, and the DLPFC is most frequently targeted in previous tDCS^11,13,23–27^ and repetitive transcranial magnetic stimulation (rTMS)^66,67^ studies of major depression. Results from this trial demonstrated (a) delivery of tDCS at the left DLPFC target with both HD and conventional montages, and (b) persistent longitudinal changes in CBF in the left DLPFC and ACC following tDCS-treatment. Notably, quality control measures including: (1) blinding, where no significant differences in Active/Sham guesses were observed between groups, and (2) electrode placement accuracy, which was measured to be within 1 cm of the stimulation-target on average across participants (within the recommendations of recent studies^68,69^), indicate integrity of the acquired data.

### Primary outcomes

Magnetic field measurements were used to investigate the delivery of tDCS currents at the left DLPFC target (hypothesis#1). The approach used here is based on Ampere’s law which states that electric currents induce linearly proportional magnetic fields. These induced fields can be detected in vivo using recently developed MRI techniques^53,57,61,70,71^. In our experiments, DE-EPI MRI revealed significant tDCS current-induced magnetic fields for both Active-stimulation montages in regions encompassing the left DLPFC (BA 46 and BA 9). Recent studies have compared magnetic field measurements and simulations and found good agreement^53,57,61,71^. However, there are still concerns regarding simulations (e.g. the assumption of tissue-conductivity values that vary across participants^72–74^), which is why we opted for magnetic field measurements in this study. While most studies at present rely on simulations (that are verified to an extent^75,76^), our DE-EPI MRI results provide verification of tDCS delivery at the stimulation-target in real-time.

Next, brain-wide longitudinal changes in CBF between Active and Sham groups were investigated using pCASL MRI (hypothesis#2). Consistent with our predictions of increased CBF based on previous studies^77,78^, measurements revealed significant increases in CBF in the Active-stimulation group (versus Sham) in two clusters. One cluster was observed in BA 46, a region confirmed to be targeted from magnetic field measurements. Moreover, when compared to the stimulation-target coordinates, the voxel with peak signal was found to be located directly underneath the stimulation-target (Fig. 4, legend). The second cluster was located in the reciprocally connected ACC. A complementary analysis using anatomical-ROI’s showed similar results (Supplementary Fig. S7). Our findings of increased CBF in these depression-relevant regions indicate that functional changes occur as a consequence tDCS-treatment. However, we cannot rule out that structural changes resulting from stimulation may contribute to the observed effects. Overall, our findings confirming delivery of tDCS at the left-DLPFC and modulation of the left-DLPFC and reciprocally connected ACC provide neurobiological evidence for systems-level changes in brain regions theorized to be dysfunctional in patients with depression.

### Montage-specific effects of tDCS

Overall, our results suggest that active HD-tDCS treatment could induce larger CBF changes compared to active-conventional in the ACC and sgACC, both part of prefrontal-limbic circuitry widely implicated in the pathophysiology of MDD^17,79^ (ACC: *d*_HD_ = 1.06 > *d*_Conv_ = 0.64; sgACC: *d*_HD_ = 0.90 > *d*_Conv_ = −0.26, Fig. 4). Since the HD montage induces electric currents that are more focal compared to the conventional montage^19,20^, an observation of comparatively stronger effects in distal brain regions with HD indicates the spread of neuromodulatory effects through a mode other than the applied electric currents, likely brain networks (as suggested by Fox et al. ^80^). Current theories of MDD attribute changes in mood and emotion in part to hypoactive mood-regulating prefrontal-limbic networks that include the DLPFC and ACC^17,18,79^, and other antidepressant treatments are shown to modulate these networks^18,37,38,40^. Our results are also consistent with the triple-network theory^81^, which posits that psychopathology in depression and other related neuropsychiatric disorders arises from dysfunction in one or more of three core networks. These networks include the central executive network (CEN), the salience network (SN), and the default mode network (DMN), and our CBF results show that regions or nodes constituting all three of these networks (i.e. the left DLPFC, ACC, and sgACC, respectively) are significantly modulated by the HD montage. Overall, the pattern of larger neurophysiological modulation by HD-tDCS suggests that HD-tDCS could be better than conventional-tDCS in modulating the CEN, SN, and DMN networks affected in depression.

### Exploratory analyses

Analysis of HDRS scores revealed non-significant differences between Active-stimulation and Sham conditions. Similar findings were observed in a recent clinical trial using the same 7 × 5 cm F3/F8 conventional montage^13^. In contrast, a significant improvement in the SHAPS score was observed in the Active-HD group compared to Sham, suggesting that particular clinical features of depression may improve with tDCS. No significant correlations between HDRS/SHAPS changes and CBF changes were observed (Supplementary Fig. S11). However, this could be due to sample size and/or because left DLPFC tDCS treatment has been shown to have a more protracted clinical effect where clinically meaningful differences are observed only after 8–10 weeks^11^. Note that our analyses of mood scores are exploratory, since we were not powered apriori to investigate efficacy of left DLPFC tDCS in this study.

The BOLD signal results from changes in blood oxy/deoxyhemoglobin concentration, and is a surrogate marker of changes in neuronal activity. An increase in neuronal activity leads to a decrease in the local oxyhemoglobin concentration, which is counteracted by an increase in blood flow. This leads to an overall increase in blood oxygenation and as a result, an increase in the measured BOLD signal^82–85^. The tDCS-induced BOLD-signal analysis was motivated by a recent proof-of-concept publication^53^ that showed BOLD-signal changes under the stimulation electrode concurrent with stimulation. In the present study, BOLD signal increases concurrent with tDCS stimulation were observed in a cluster in BA 46 (an area confirmed to be targeted by magnetic field measurements) in the Active-HD group compared to Sham. These changes were at least an order of magnitude larger than potential confounds (calculated using the method of Jog et al. ^53^ and described in Supplementary Fig. S12). Although exploratory, our findings of tDCS-induced neurophysiological changes near the stimulation-target concurrent with stimulation complement our main findings demonstrating targeting and CBF-modulation of the left-DLPFC stimulation-target by tDCS. Further, these same BOLD-signal changes were observed to be negatively correlated with %ch-SHAPS in the Active-HD group. Notably, current-induced magnetic fields and simulated current densities from the same region were not significantly correlated with %ch-SHAPS ((*r*,*p*) = (0.23,0.42)/(−0.15,0.60), Supplementary Fig. S13). These preliminary results suggest that the tDCS-induced BOLD-signal change at baseline could have utility as a biomarker in predicting anhedonia response, and suggest a focus for future research.

### Rationale for montage and treatment parameters

In this study, we used HD and conventional montages to administer 2 mA × 20 min of tDCS at rest in depressed participants over 12 sessions. Here we discuss how specific montage and treatment parameters were selected for administering tDCS. First, the left DLPFC stimulation target was chosen because altered prefrontal-subcortical circuitry is widely implicated as contributing to the pathophysiology of depression^63–65^, and the DLPFC has been a common target in previous studies^11,13,23–27^.

To modulate the left DLPFC, two montages, a HD and a conventional montage were selected. Notably, HD-tDCS, which provides spatially focal stimulation with respect to conventional tDCS^19,20^, has not yet been the focus of any published randomized clinical trial in depression. A common 4 × 1 ring arrangement was used for the HD-montage, with the anode positioned over the stimulation-target and the four cathodes placed 5 cm away and equidistant from each other^19,20^. Our choice of the separation distance between anode and cathode electrodes in the HD-montage was supported by computational models of current-flow. That is, as shown in Supplementary Fig. S14, these simulations indicated that a separation distance of 5 cm did not overly bias one montage in terms of the delivered current-density; with both montages providing comparable current-density magnitudes at the left DLPFC stimulation-target. The conventional montage was designed to reflect a typical tDCS montage. Here, a bicephalic arrangement employing 7 × 5 cm electrodes commonly used in depression studies (as described in a review by Kuo et al. ^10^) was used.

The duration of the tDCS sessions was based on prior literature. That is, up to the inception of our study in 2017, the large majority of studies preferred 20 min of tDCS-stimulation^10^. Also based on previous studies, we elected to include 12 sessions^48–51^, including two sessions administered in the MR environment and 10 sessions administered outside the scanner. Though prior depression tDCS studies have mostly included <15 sessions^86^, some data suggests a relationship between treatment duration and clinical effects^87,88^. However, since the focus of this study was on target engagement, we hypothesized that this stimulation protocol would lead to measurable changes in our neurobiological probe (i.e. CBF).

Finally, the biophysical effects of tDCS support that neural modulation may be amplified for brain networks already functionally active^89^. Thus, including functional tasks or treatments together with tDCS administration may produce more pronounced or targeted modulation of specific brain circuits linked depression. However, results from initial studies combining tDCS with adjunctive treatments or tasks have been mixed^7^, and in depression, tDCS is usually applied without the simultaneous administration of systematically controlled functional probes or treatments, e.g. refs. ^11,13^, Consequently, for this study we chose to restrict the exploratory clinical trial to investigating tDCS administered at rest.

Note that although left anodal DLFPC tDCS has been used in a majority of depression tDCS studies^10^, other montages may better target specific neural circuits contributing to specific clinical features of major depression. For example, while anodal DLPFC tDCS may better engage dorsal forebrain-limbic systems (including the DLPFC and ACC), computational modeling has shown that bitemporal tDCS has greater engagement of deeper brain structures. Further, left DLPFC tDCS with extra-cephalic right deltoid cathode placement may induce changes in current flow in deeper ventro-limbic structures^90,91^. How these montages affect current flow and neuroplasticity in-vivo, however, remains to be explored in future studies

### Limitations

The present study was powered to detect changes in magnetic fields and longitudinal changes in CBF to confirm left-DLPFC tDCS target engagement and treatment-induced neurophysiological effects, respectively^43^. We were not powered a priori to investigate changes in BOLD-signal or clinical efficacy. Additionally, we did not have an experimental condition where tDCS was administered to a control region, and therefore cannot conclude that the observed effects are specific to left DLPFC-targeted stimulation in depression. We also did not measure CBF mid-way through the study; this could have clarified the trajectory of CBF changes, and is a limitation of our study. With regard to clinical metrics, a recent study showed that HDRS-scores only show clinically meaningful effect-size differences between Active-stimulation and placebo groups 8–10 weeks following treatment^11^. To address this, future investigations should include extended longitudinal follow-ups in larger samples.

## Conclusion

In this study, we used magnetic field and CBF measurements to (a) confirm delivery of tDCS on the DLPFC neural target, and (b) demonstrate persistent post-treatment modulation of prefrontal-limbic circuitry in major depression. Our results also indicate that HD-tDCS may induce stronger network-modulations compared to conventional tDCS. Future studies are needed to specifically investigate the clinical efficacy of HD-tDCS in depression.

## Supplementary information

Supplemental Material

## Data Availability

The data is available on the NIMH Data Archive (https://nda.nih.gov/edit_collection.html;jsessionid=6A0901D897C728332C4956C2D53A59DB.node2?id=2737&source=RDoCdb).
