# Supplementary material for: In-vivo imaging of targeting and modulation of depression-relevant circuitry by transcranial direct current stimulation: a randomized clinical trial
Source: Transl Psychiatry. 2021 Feb 24;11:138. doi: 10.1038/s41398-021-01264-3 (PMC7904813; doi:10.1038/s41398-021-01264-3)
Supplement: Supplementary file 1 — Supplemental Material [file 41398_2021_1264_MOESM1_ESM.docx]

**Supplementary materials**

**S1: Inclusion/exclusion criteria**

**S2: Between-group clinical/demographic characteristics, differences in electrode-placement and active/sham guesses**

**S3: Electrode placement procedure**

**S4: DE-EPI applied-current block design and MRI sequence parameters**

**S5: Preprocessing of imaging-data**

**S6: Stray field correction for magnetic field measurements**

**S7: Complementary analysis of rCBF extracted from anatomical regions (DLPFC-BA 46 and dACC)**

**S8: MNI co-ordinates of all significant clusters**

**S9: Stimulation related subject discomfort**

**S10: Linear mixed-model analysis of the clinical scores**

**S11: Correlation between %ch-HDRS and SHAPS and measured CBF changes.**

**S12: Potential tDCS-induced confounds in the BOLD signal near the stimulation target**

**S13: Correlation between %ch-SHAPS and measured current induced magnetic field, and simulated current density.**

**S14: Separation distance between the anode and cathode electrodes of the HD-montage**

**S1: Inclusion/exclusion criteria**

Exclusion criteria included (a) pregnancy, (b) non-English speaking, (c) substance use disorder within last 12 months, (d) neurological conditions associated with brain abnormalities (e.g. traumatic brain injury, recent stroke, tumor), (e) any contraindication to tDCS (e.g. skin disease or treatment causing irritation), (f) any condition that would contraindicate MRI (metal implants, claustrophobia or a breathing or movement disorder), (g) currently receiving any form of cognitive behavioral therapy, dialectical behavioral therapy, or acceptance and commitment therapy, (h) change in antidepressant medication within 6-weeks of starting the trial, (i) severe or treatment resistant depression – HAMD scores > 24 and a history of a major depressive episode lasting >2-years or failure to 2 or more antidepressant trials in the current index episode, (j) any neuromodulation therapy (e.g., ECT, rTMS, DBS, VNS or tDCS) within the last 3-months, (k) current or past (within the last 1-month) use of anticonvulsants, lithium, psychostimulant, dexamphetamine, (l) current use of decongestants or other medication including sleeping aids previously shown to interfere with cortical excitability, (m) diagnosis of schizophrenia axis I disorder, or dementia of any type, (n) bipolar I disorder (due to possible risk of mania and because lithium and anticonvulsants are excluded), (o) diagnosis of seizure disorder or history of seizures, (p) depression related to serious medical illness (i.e., mood disorder due to general medical condition), (q) actively suicidal as defined by a score of 4 on item 3 of HAMD, and (r) missing more than 2 study visits. Based on the Structured Clinical Interview for DSM-5 ([1](#_ENREF_1)), in addition to major depressive disorder, comorbid diagnoses included panic disorder n=5, generalized anxiety disorder n=11, other specified anxiety disorder n=3, social anxiety disorder n=7, other specified eating disorder n=1, adult ADHD n=1, posttraumatic stress disorder n=12, other specified bipolar disorder n=1.

**S2: Between-group clinical/demographic characteristics, differences in electrode-placement and active/sham guesses**

Table S shows clinical and demographic characteristics of the enrolled participants for the Sham, Active-Conv, Active-HD groups, and overall. Each characteristic was tested for differences between groups using a 1-way ANOVA for continuous data (and 𝜒^2^-test test for categorical data), and the p-values are reported in the last column. No significant differences in clinical and demographic characteristics were observed.

**
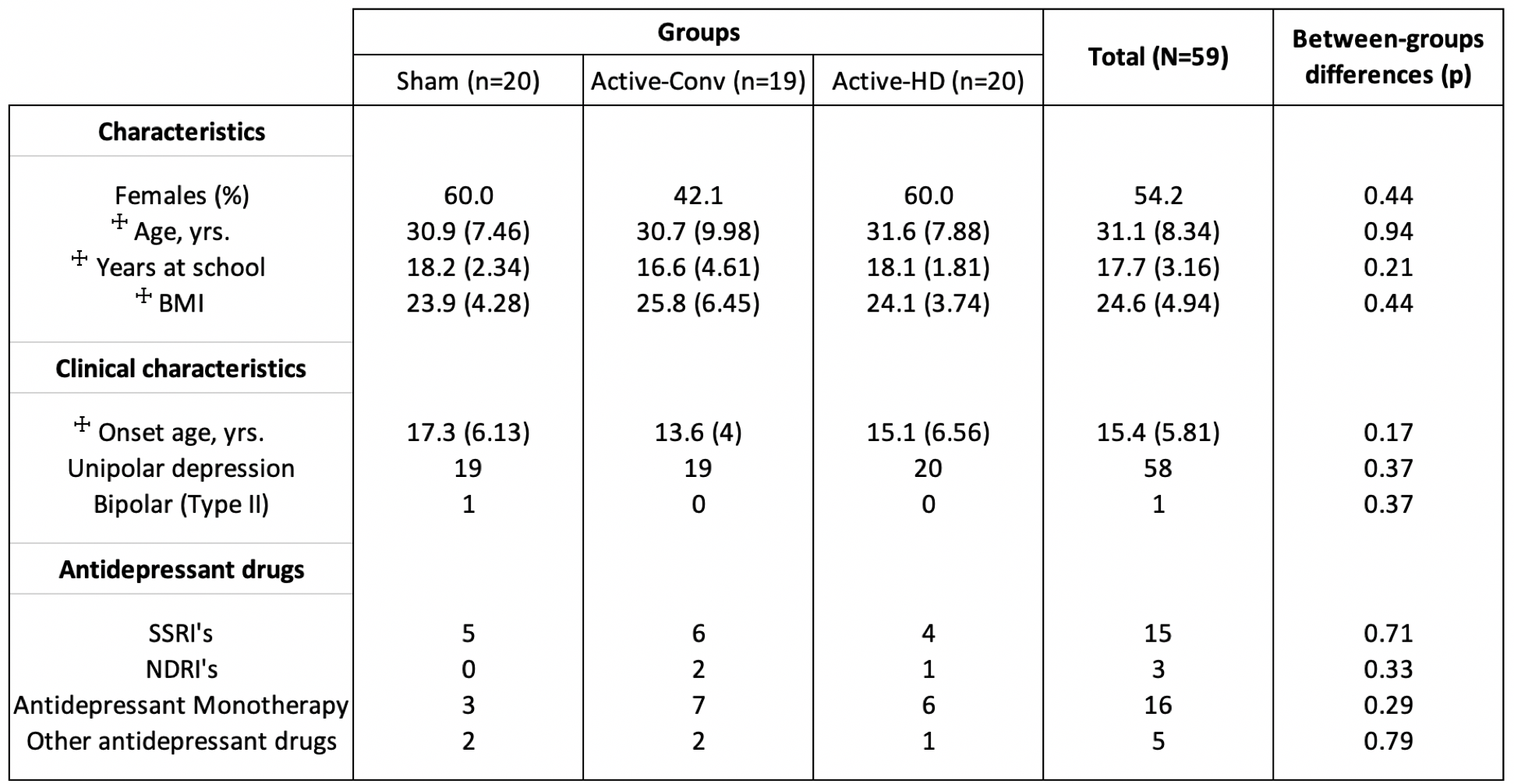
**

**Table S2: Clinical and demographic characteristics.** Characteristics of the enrolled participants are shown by group (columns 1-3) and overall (column 4). No significant differences between any characteristics were observed (tested with 1-way ANOVA for continuous, and 𝜒^2^-test for categorical random variables, p-values shown in column 5). ☩ are reported as Mean (SD), and total-counts are reported for the rest. SSRI=Selective Serotonin Reuptake Inhibitor, NDRI=Norepinephrine Dopamine Reuptake Inhibitor. Other antidepressants include serotonin noradrenaline reuptake inhibitors (SNRIs), monoamine oxidase inhibitors (MAOIs) and tricyclic antidepressants (TCAs). Participants using anticonvulsants, lithium, psychostimulant and dexamphetamine were excluded, as were participants with bipolar I disorder (due to possible risk of mania and because lithium and anticonvulsants were also exclusionary criteria). The full inclusion/exclusion criteria are described in Methods and S1.

Electrode-displacements measured using the Brainsight neuronavigation system([2](#_ENREF_2)) at the mid-trial visit (visit 6) were recorded, and tested for differences between groups using a 1-way ANOVA. No significant differences were observed in electrode-displacements between Sham, Active-Conv and Active-HD groups (p = 0.81; Mean ± SD; [Sham]: 7.5 ± 3.5 [HD]: 7.1 ± 2.0; [Conv]: 6.8 ± 3.1; [Overall]: 7.0 ± 2.8, max-displacement = 13, all measurements in millimeters).

After each participant’s final treatment-session, both participants and assessors were polled as to whether they thought the administered treatment was active (yes/no), and their confidence in their answer (scale 1-10). After binning by high and low confidence, the number of active/sham guesses were calculated across groups, and compared using a $\chi$^2^ test. No significant differences were observed between Sham, Active-Conv and Active-HD groups (Participants: $\chi$^2^=1.54, p=0.46; Assessors: $\chi$^2^=0.045, p=0.97).

**S3: Electrode placement procedure**

EASY caps([3](#_ENREF_3)), premarked with 10-20 EEG locations, were utilized for electrode placement. For each participant, a cap-size that the participant found comfortable was chosen, and placement was performed as follows: first, the cap was secured with a chinstrap which was marked so the cap could be secured with the same tension every session. Next, the cap was adjusted such that the mid-point of the Fp1/Fp2 line was 10% of the inion-nasion distance, following which the cap was further adjusted such that the L/R tragus to T7/T8 distances were identical. The cap was then secured by taping it down over the participant’s upper cheeks. The nasion to Fp1/Fp2 line and L/R tragus to T7/T8 distances were recorded. For all subsequent sessions, the cap was placed to match these distances to within 2 mm. Note that the correspondence of the reference points to the actual Fp1/T7 locations etc. is not important; what is crucial for correct placement is to ensure that the distances of reference points from anatomical landmarks match the recorded distances to within 2 mm.

Next, the Brainsight neuronavigation system([2](#_ENREF_2)) was used to mark the stimulation target on the cap. The location of the stimulation target was calculated in the participant’s native T1 coordinate-space by transforming the stimulation-target coordinate ([-46, 44, 38]_MNI_) to the participant’s T1 (acquired during visit 0). This was achieved using inverse normalization (SPM12), followed by shortest-distance projection of the coordinates from the cortical location onto the scalp. Next, Brainsight was used to map the participant’s T1 to the participant using the nasion and L/R tragus points; after which the stimulation target was marked onto the participant’s cap. For all subsequent tDCS sessions, electrode placement was performed by identifying the stimulation site through placement of the now-marked cap; the placement being performed using the procedure described in the previous paragraph.

**S4: DE-EPI applied-current block design and MRI sequence parameters**

The DE-EPI scan is 12.5 minutes long. During the scan, electric current (for the ‘Active’ group) was applied in real-time using a block-design as shown in Figure S4 below.


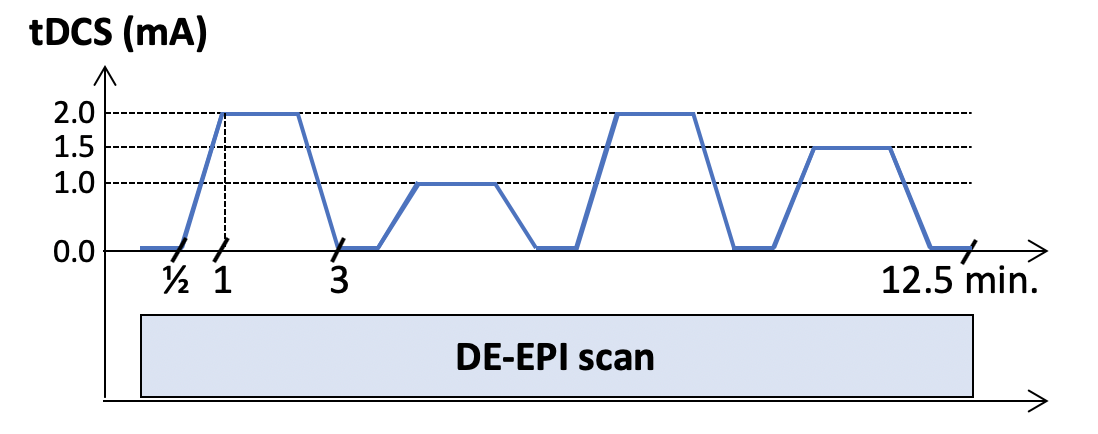


**Figure S4: DE-EPI Block-design.** During the DE-EPI scan, tDCS electric current was applied in a block-design, with 30 sec. ramp-times, 1.5 minutes of “on” current and 0.5 minutes of “off” current. The current-intensities were varied as shown in the figure (2, 1, 2, 1.5mA for blocks 1-4 respectively).

For the Sham group, current was ramped up to 2mA, and then ramped down at the beginning of the scan. All ramp times were 30 sec. Sequence parameters for the DE-EPI scan were: TE1/TE2 = 11/26 ms, TR=2s, 90^0^ FA, 3.4x3.4x4 mm^3^ voxel, 64x64 matrix, 35 slices (ascending), 2365 Hz/Px bandwidth, 7/8 partial Fourier, R=2 GRAPPA acceleration, N = 2 back-to-back scans (data was averaged post quantification). Note that sequence parameters and block-design are identical to those reported in a recent technique-validation study([4](#_ENREF_4)).

Sequence parameters for the pCASL scan were TR/TE=4000/25ms, 96x96 matrix, 2.5x2.5x2.5mm^3^, 48 slices, labeling duration=1.5sec, post-labeling delay = 1.8s; N = 2 back-to-back scans (data was averaged post CBF-quantification).

The T1-MPRAGE sequence parameters were identical to those utilized in the Human Connectome Project([5](#_ENREF_5)), and are TE1/TE2/TE3/TE4 = 1.81/3.6/5.39/7.18 ms, TI=1s, TR=2.5s, 8^0^ FA, 0.8x0.8x0.8 mm^3^ voxel, 320x320 matrix, 208 slices, 740 Hz/Px bandwidth for all TE’s, 6/8 partial Fourier, R=2 GRAPPA acceleration.

All scans were acquired with a 64ch-head coil on a Siemens 3T PRISMA scanner.

**S5: Preprocessing of imaging data**

DE-EPI: Acquired phase volumes were unwrapped using a 4D region-growth algorithm([6](#_ENREF_6)) (3 spatial dimensions, and 1 temporal dimension). Next the phase data at the 2^nd^ echo was realigned to the first phase-volume using SPM12, converted to magnetic field measurements by dividing by the TE and gyromagnetic ratio of protons, and finally modeled using a general linear model with the applied current as predictor. Nuisance regressors included motion parameters and the global signal. The slope of this fit can be interpreted as the tDCS current-induced magnetic field. The phase data at the first echo was unused. The MRI-magnitude data at the 2^nd^ echo encodes the BOLD-signal, and was similarly realigned to the first volume using SPM12, and modeled using a general linear model with the applied current (convolved with the hemodynamic response function) as a predictor and 24 motion-parameter regressors([7](#_ENREF_7)). Both tDCS-current induced (a) magnetic field maps and (b) BOLD-signal change maps were coregistered to the T1 structural scans and normalized to the MNI atlas space using SPM12 for group-level statistics. Note that this preprocessing is as described in a recent technique validation study([4](#_ENREF_4)).

pCASL data: The acquired pCASL data was motion corrected using SPM12, and in-house processing scripts (based on ([8](#_ENREF_8))) were used to quantify CBF maps. Next, CBF maps were coregistered to the structural scans, normalized to MNI space, masked using a gray matter mask (SPM12 TPM mask, thresholded at 0.2) and smoothed using a 7.5mm FWHM (i.e. 3 voxel wide) gaussian kernel in preparation for group-level statistics.

**S6: Stray field correction for magnetic field measurements**

As shown in ([9](#_ENREF_9), [10](#_ENREF_10)), measured current induced magnetic fields could be affected by stray fields induced by the current carrying wires. In our experiments, a cable coating similar to ([9](#_ENREF_9), [10](#_ENREF_10)) was used that made the current-carrying wires visible. The imaged wires were segmented using ITK-SNAP([11](#_ENREF_11)) and the obtained coarse segmentations were smoothed using a geometric-centroid interpolation algorithm. The obtained wire-paths were used to calculate the induced magnetic field using the Biot-Savart law implemented in the Biot-Savart magnetic toolbox (MATLAB, ([12](#_ENREF_12))) :

$$\boldsymbol{B}=\frac{\mu_{0}}{4\pi}\int\frac{I\boldsymbol{dl}\times\boldsymbol{r}}{\boldsymbol{r}^{\boldsymbol{3}}}$$

Here, vector quantities are in bold. B is the magnetic field calculated at the position r (relative to the current-element), $\mu_{0}$ is the magnetic permeability (constant), I is the electric current, and dl is the current-element (of length 0.1mm). The calculated fields were projected along the MRI static-field (ie. Bz) direction, after which average fields in the BA 46 and BA 9 ROIs (Sallet atlas([13](#_ENREF_13))) were calculated and subtracted from measurements.

**S7: Complementary analysis of CBF extracted from anatomical regions (BA 46 and dACC)**

To additionally support the voxelwise CBF results, a complementary analysis of the CBF data was performed using ROIs from anatomical atlases. Average CBF-values in the BA 46 and dACC ROIs were extracted using ROIs from the Sallet([13](#_ENREF_13)) and FreeSurfer-Destrieux([14](#_ENREF_14)) atlases respectively. These were modeled using a general linear mixed model (GLMM) with predictors including tDCS condition, time, and the tDCS condition by time interaction. Finally, post-hoc t-tests were used to quantify the effect sizes and significance of change in CBF in Active-stimulation conditions compared to Sham. Shown in Figure S7, the results are similar to the voxel-results although lower in significance (which is expected, since the ROIs are large). In both regions, a significant time X condition interaction was observed. Posthoc t-tests showed significant CBF changes with Active-HD, and non-significant trending effects with Active-Conv in BA 46 and dACC respectively. In BA 46, both montages showed medium-to-large effect sizes, while in dACC, Active-HD showed a large effect-size compared to Active-Conv, similar to the voxelwise results (BA 46: [HD] *d*=0.72, p=0.031; [Conv] *d*=0.52, p=0.13; dACC: [HD] *d*=0.84, p=0.013; [Conv] *d*=0.60, p=0.086).


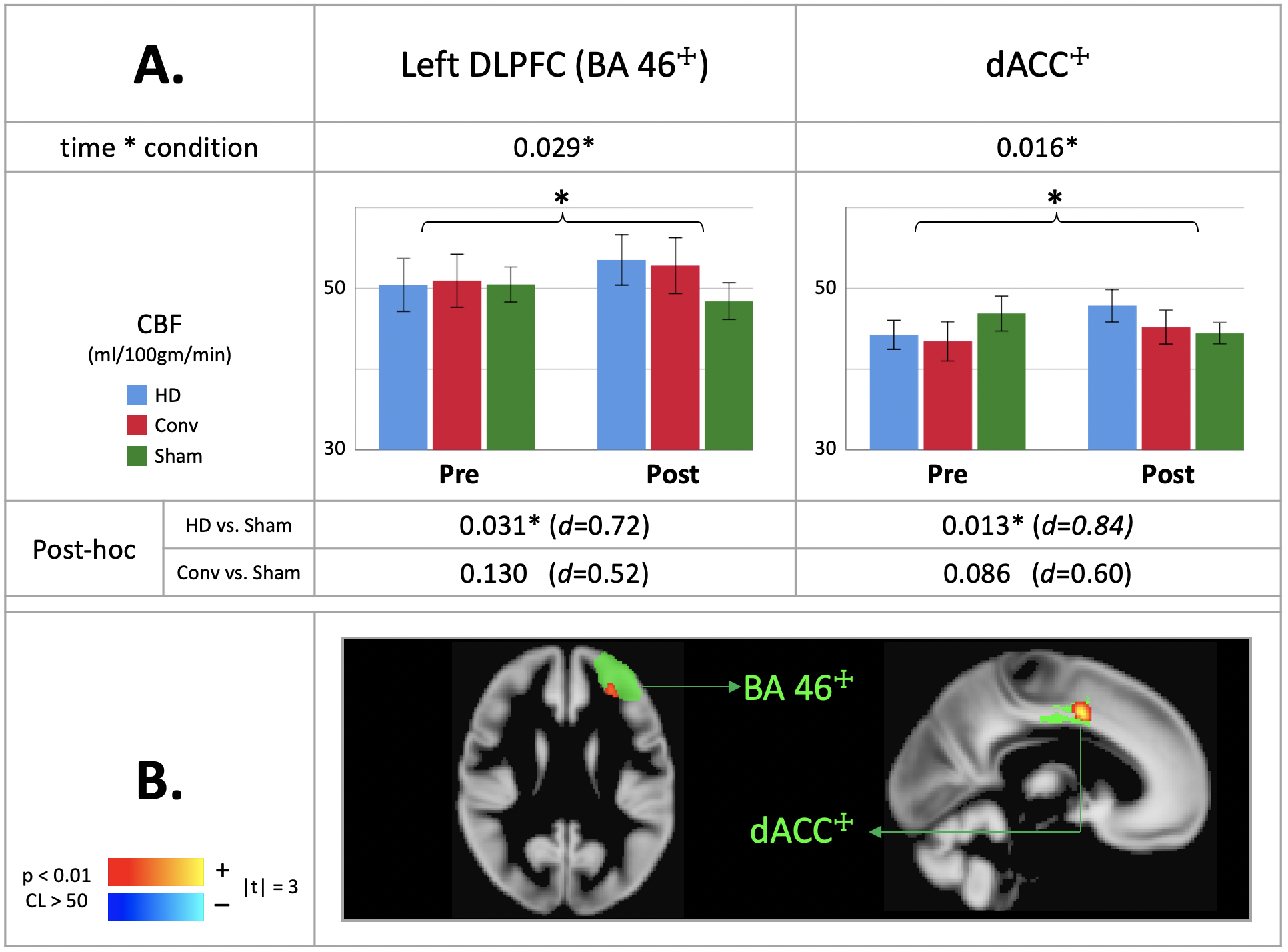


**Figure S7: Complementary analysis of rCBF extracted from anatomical regions. A.** The bar graphs show average CBF values in the BA 46 and dACC ROIs before and after the 12-day tDCS trial. Indicated on these bar graphs are significant time by tDCS condition interactions. The middle rows show p-values and effect sizes (Cohen’s d) for post-hoc tests comparing change in average CBF. These results indicate modulation of CBF in the DLPFC and ACC by tDCS over time. **B.** shows the voxelwise results of Figure 4.A overlaid on the BA 46 and dACC ROIs (from the Sallet([13](#_ENREF_13)) and FreeSurfer-Destrieux([14](#_ENREF_14)) atlases respectively), with the ROIs shown in green.

**S8: MNI co-ordinates of all significant clusters**

Here, we report significant clusters for all voxel-wise analysis performed in the study.

1. For the CBF data, the clusters reported in the main manuscript were the only clusters that were observed.

2. For the tDCS-induced BOLD-signal change data, the following clusters were observed:


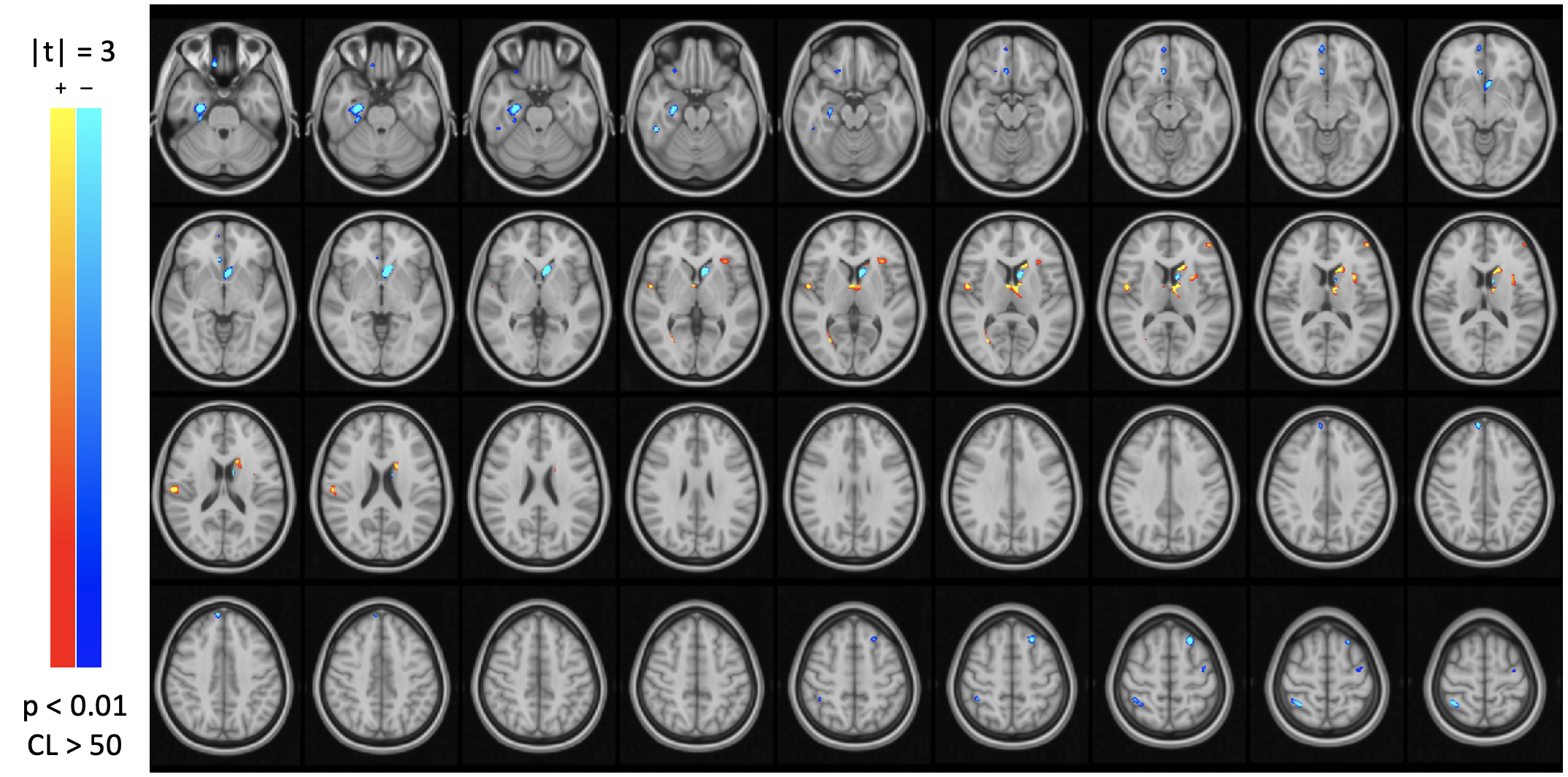


**Figure S8: tDCS-induced acute BOLD-signal changes (Act > Sham; p < 0.01, cluster-size > 50)**

As seen in the figure, most of the clusters where activity increased with stimulation were in the targeted left hemisphere, including one cluster at [-45, 45, 12] in BA 46 (a region confirmed to be targeted from magnetic field measurements). Conversely, clusters in which activity decreased with stimulation were primarily in the right hemisphere. The full list of significant clusters is listed in the table below.

**Table S8: Locations of significant clusters in the tDCS-induced acute BOLD-signal (Act > Sham).**


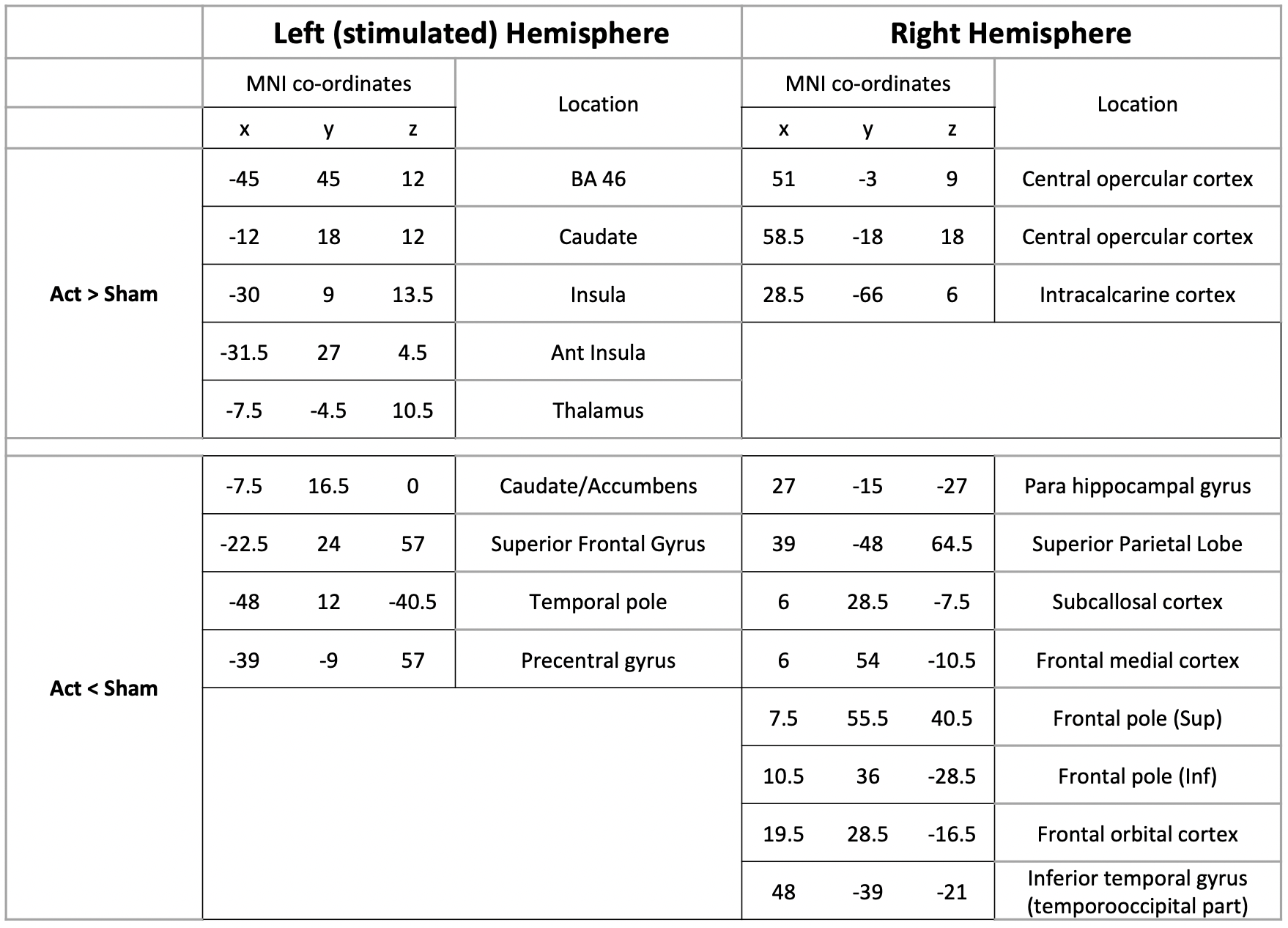


**S9: Stimulation related subject discomfort**

Stimulation related subject discomfort was assessed using the GASE (Generic Assessment of Side Effects) scale ([15](#_ENREF_15)). The GASE data was acquired after every study visit for each subject. Items relevant to assessing stimulation-related discomfort (including headache, dizziness, palpitations, breathing difficulty, nausea, rash, fever, and fatigue) were averaged across visits for each subject, and added to calculate a modified GASE score. Shown in Figure S9, no significant differences between the sham, active-conventional and active-HD groups were observed (p=0.20).

**
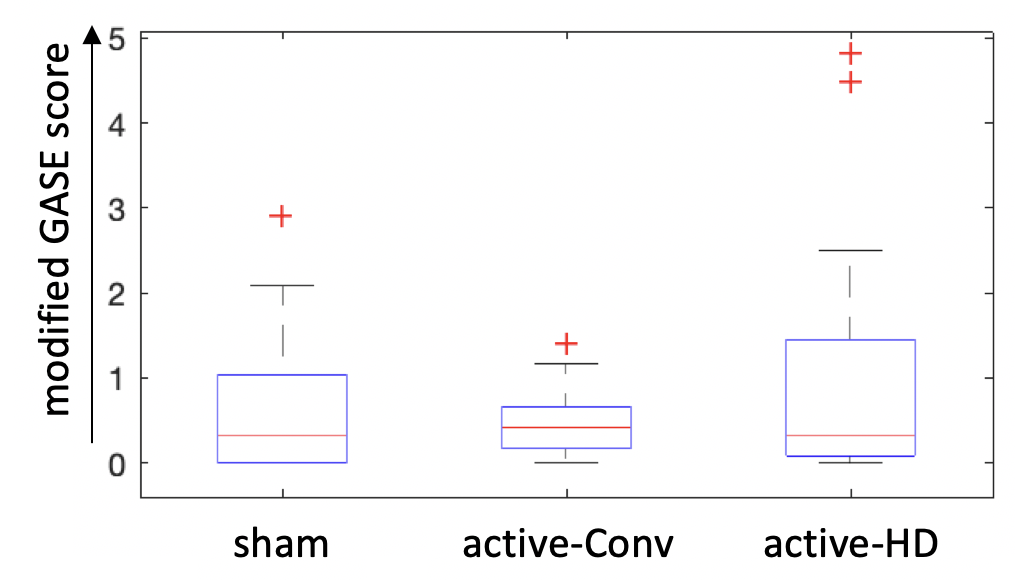
**

**Figure S9: Stimulation related subject discomfort.** The box plot shows stimulation-related subject discomfort for the sham, active-conventional and active-HD groups measured using a modified GASE score (see text for details). The red lines and edges of the box indicate median discomfort and 25/75 percentiles respectively. Two subjects in the active-HD group were observed to feel more discomfort compared to the rest. Even so, no systematic difference between groups was observed with a 1-way ANOVA using all of the data (p = 0.20). When the outliers were excluded, systematic differences between groups remained non-significant (p=0.54).

**S10: Linear mixed-model analysis of the clinical scores.**

A linear mixed effect model was also used to analyze the clinical scores, and did not reveal significant results, though it should be noted that this analysis was exploratory, and we were not powered to investigate potential time*condition interaction effects in clinical scores.

**
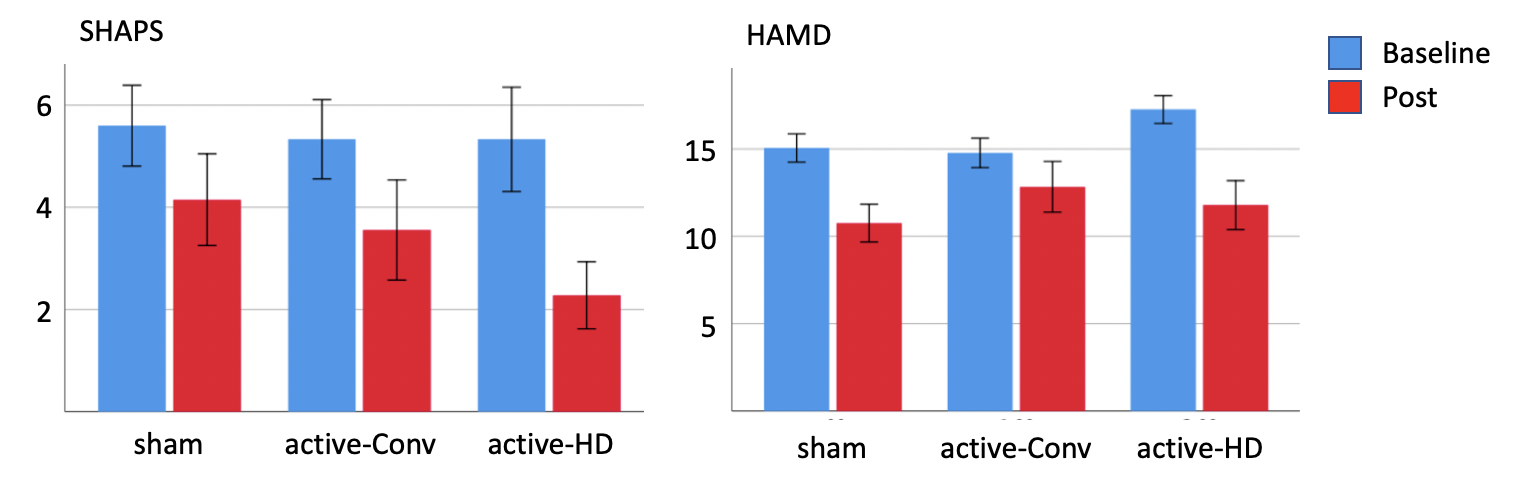
**
**Figure S10: Mixed-analysis of the clinical scores.** Box plots show the SHAPS and HAMD scores at baseline and post-treatment for each of the 3 groups. Mixed analysis did not reveal significant effects (p = 0.42 and 0.19). The response rate was 30%, 26.3% and 40% in the sham, active-conv and active-HD groups respectively, and the remission rate was 25%, 10.5% and 25% for the same (responders defined as %subject whose HAMD scores improved by >50%, and remitters defined as %subjects whose post-treatment HAMD was <= 7).

**S11: Correlation between %ch-HDRS/SHAPS and measured CBF changes.**

As shown in Fig S11, no significant correlations were observed between %ch-HDRS/SHAPS and the CBF changes in each of the three ROIs. However, this could be because the left DLPFC tDCS treatment has been shown to induce clinically significant effect-sizes 10 weeks post treatment, which is much later than our planned acquisition at the post-treatment 2-week time-point. This is a limitation of this study, and is discussed in the limitations section in the main manuscript.


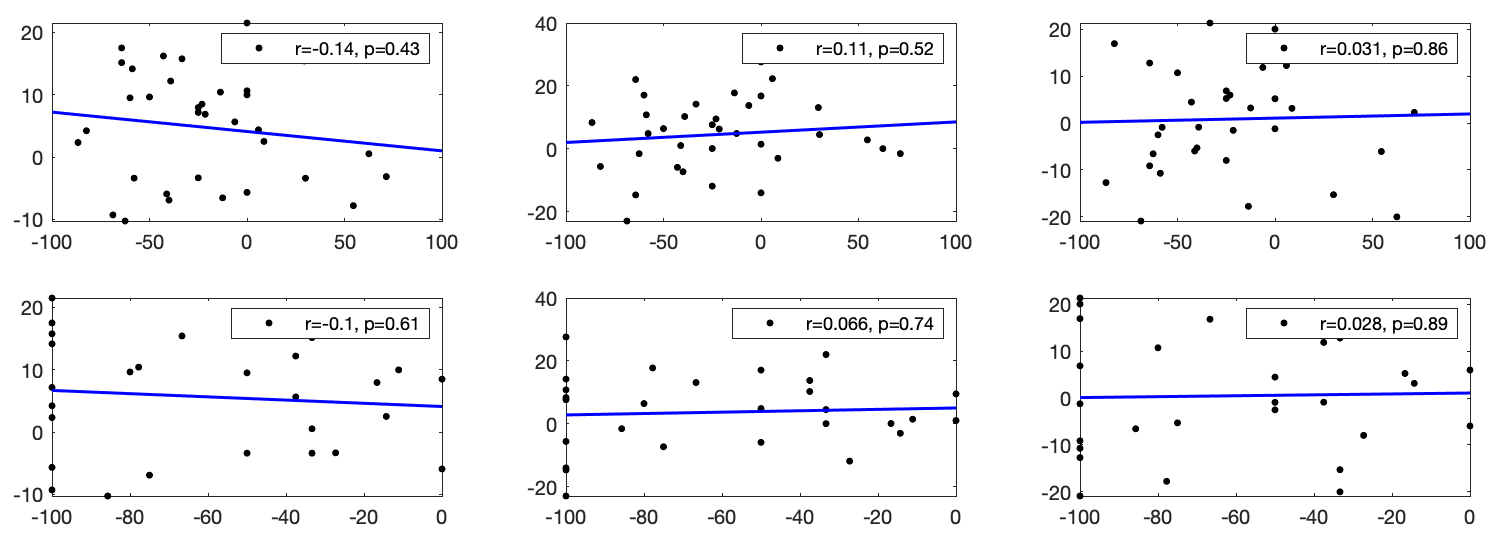


**Figure S11: Correlation between %ch-HDRS/SHAPS and measured CBF changes.** Rows correspond to %ch-HDRS and %ch-SHAPS, while columns correspond to the left DLFPC, ACC and Sg. ACC regions shown in Fig 3. Overall, no significant correlations between CBF changes and %ch-HDRS/SHAPS were observed.

**S12: Potential tDCS-induced confounds in the BOLD signal**

tDCS currents induce magnetic fields along the MRI static field Bz. The resulting magnetic field inhomogeneity could potentially induce confounds in the BOLD signal. Here, we used the models of ([4](#_ENREF_4)) to estimate the size of tDCS-induced confounds, and compared it to the measured tDCS-induced BOLD signal change near the stimulation target.

To estimate the confound size, we first calculated the gradient of the measured current-induced magnetic field (|▽B_z_|), following the approach of ([4](#_ENREF_4)). |▽B_z_| was calculated voxel-wise for each participant in the ROI where the significant tDCS-induced BOLD-change was measured (ROI shown in Figure 3.B). Next, these estimates were plotted in a histogram for each Active montage. Shown in Figure S12, from these distributions, a worst-case value of the magnetic field inhomogeneity (|▽B_z_|) = 3nT/mA tDCS was selected.


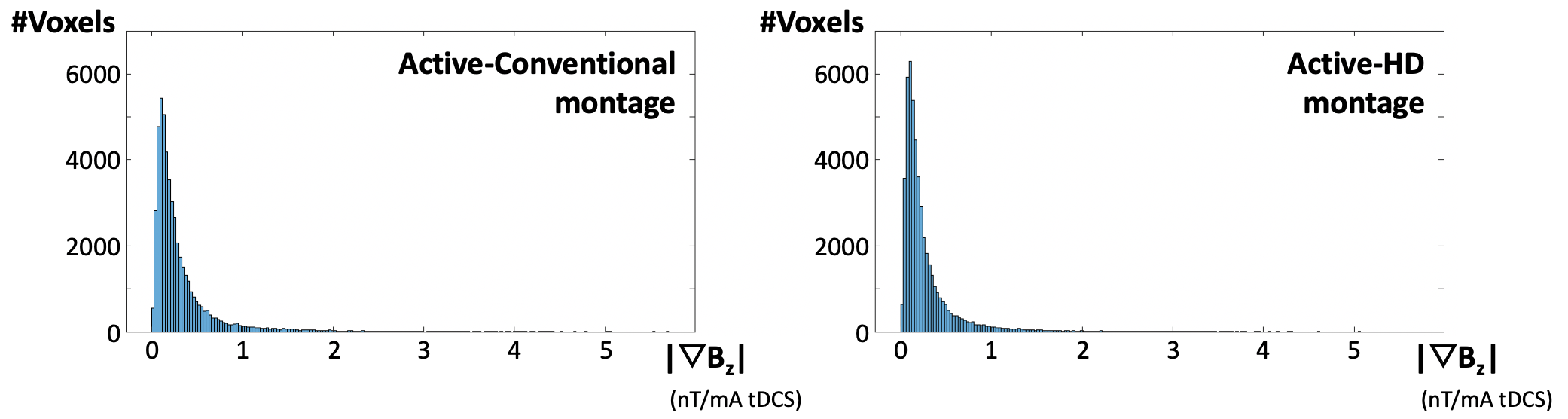

**Figure S12: Magnetic field inhomogeneity (|▽B_z_|) in the significant cluster at [-45,45,12] (shown in Figure 3.B) in the Active-Conventional and Active-HD montages.** |▽B_z_| was calculated for each voxel in this cluster, pooled over participants, and plotted as shown in the histograms. A worst-case value of |▽B_z_| = 3nT/mA tDCS was used to calculate the tDCS-induced BOLD-confound (described below).

Using the worst case value, confound sizes were estimated using the two models described in ([4](#_ENREF_4)). Note that because the confound estimates are relative to the average signal in the voxel, these values were compared to the observed tDCS-induced BOLD signal change divided by the average BOLD-signal (obtained from the intercept-term in the modeled BOLD-data). Shown in Table S12, the estimated confounds were at least an order of magnitude smaller than the observed BOLD-signal changes; indicating that the observed signal-changes likely reflect neurophysiological changes.


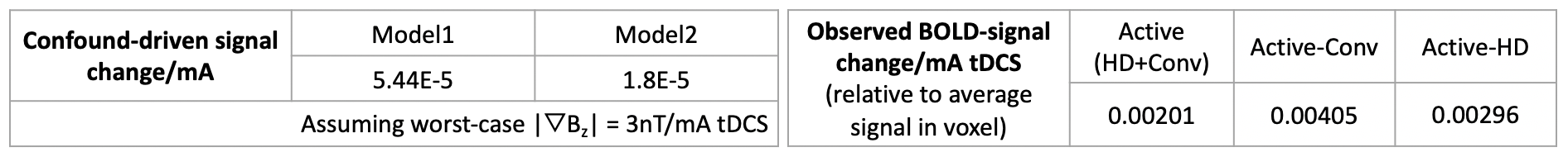

 **Table S12: tDCS-induced confounds in the BOLD signal.** Confounds were estimated using a worst case value of the field inhomogeneity |▽B_z_| (= 3nT/mA tDCS) and the models of ([4](#_ENREF_4)). These values were compared with the observed BOLD-signal changes/mA tDCS in the significant cluster at [-45,45,12] (shown in Figure 3.B) relative to the average signal in the voxel (obtained from the intercept of the modeled BOLD-data). As can be seen, the estimated tDCS-induced confounds are an order of magnitude smaller than the measured changes.

**S13: Correlation between %ch-SHAPS and the measured current induced magnetic field and simulated current density.**

Because we observed significant differences between Active-HD and Sham groups in %ch-SHAPS and the baseline tDCS-induced BOLD-signal change near the stimulation target, we investigated these two metrics for correlations; the idea being that significant correlations could indicate a biomarker of anhedonia-response for future research.

A significant negative correlation (i.e. the larger the tDCS-induced BOLD signal at baseline near stimulation target, the greater the anhedonia improvement) was observed, as shown in Figure 5.B. In other words, we observed a correlation between a marker of the brain’s response to stimulation, and mood-improvement. Here, we investigated whether the mood-improvement was also correlated with measures of tDCS current i.e. the measured tDCS current-induced magnetic field (shown in Figure 3), or the simulated current-density from the same region. The motivation here was to explore the characteristics of the BOLD biomarker; no correlations in this exploratory analysis could potentially indicate *unique* utility of the BOLD biomarker and provide motivation for future research.

The measured tDCS current-induced magnetic fields in the BOLD-ROI (ROI shown in Figure 3.B) were extracted for each participant and correlated with %ch-SHAPS. For the simulations, the acquired T1-MPRAGE’s were segmented using SIMNIBS([16](#_ENREF_16)). Next, default tissue conductivities provided in SIMNIBS (from ([17](#_ENREF_17), [18](#_ENREF_18))), were used, and simulations of the current-density were performed. Finally, average current-densities for each participant in the BOLD-ROI were extracted and correlated with %ch-SHAPS. As shown in Figure S13 below, no significant correlations were observed.


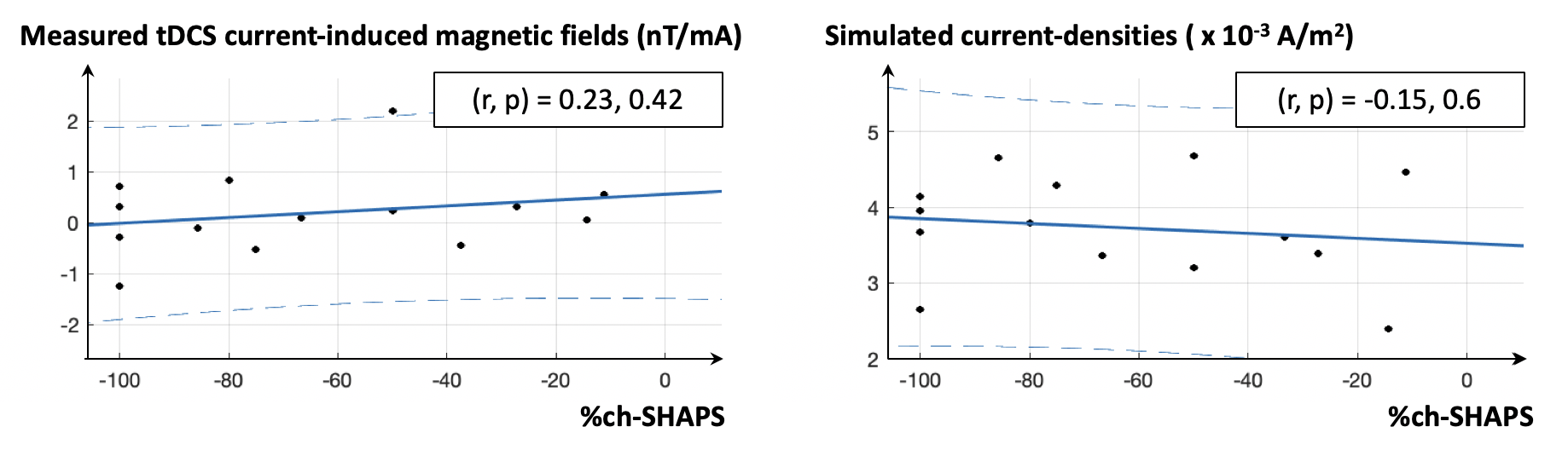


**Figure S13: Correlations of %ch-SHAPS with current-induced magnetic fields and simulated current-densities from the BOLD-ROI (shown in Figure 3.B)**. No significant correlations were observed between %ch-SHAPS and the (a) tDCS current-induced magnetic field, (r,p)=(0.23, 0.42), or the (b) simulated current-densities, (r,p)=(-0.15, 0.6) .

**S14: Separation distance between the anode and cathode electrodes of the HD-montage.**

A common 4x1 ring arrangement was used for the HD montage, with the anode positioned over the stimulation target, and the 4 cathodes placed 5cm away and equidistant from each other ([19](#_ENREF_19), [20](#_ENREF_20)). The choice of the 5cm separation distance was also supported by computational models of current flow. Here, conventional and HD tDCS montages, the latter with 3, 5, 7, 9 and 11 cm separation, were modeled on a standard MNI-template head (1mm resolution) using SIMNIBS([16](#_ENREF_16)). Next, default tissue conductivities from ([17](#_ENREF_17), [18](#_ENREF_18)) were used, and simulations of the current-density were performed in the left DLPFC ROI. As shown in Fig S14, the HD-montage with a 5cm separation distance induced current-density magnitudes that were comparable to the conventional montage (**S14**). Thus, this separation distance was selected since it did not overly bias one montage over the other in terms of the magnitude of current density. Note that at the whole-brain level however, the HD montage is more spatially-specific than the conventional montage by design ([19](#_ENREF_19), [20](#_ENREF_20)).

**
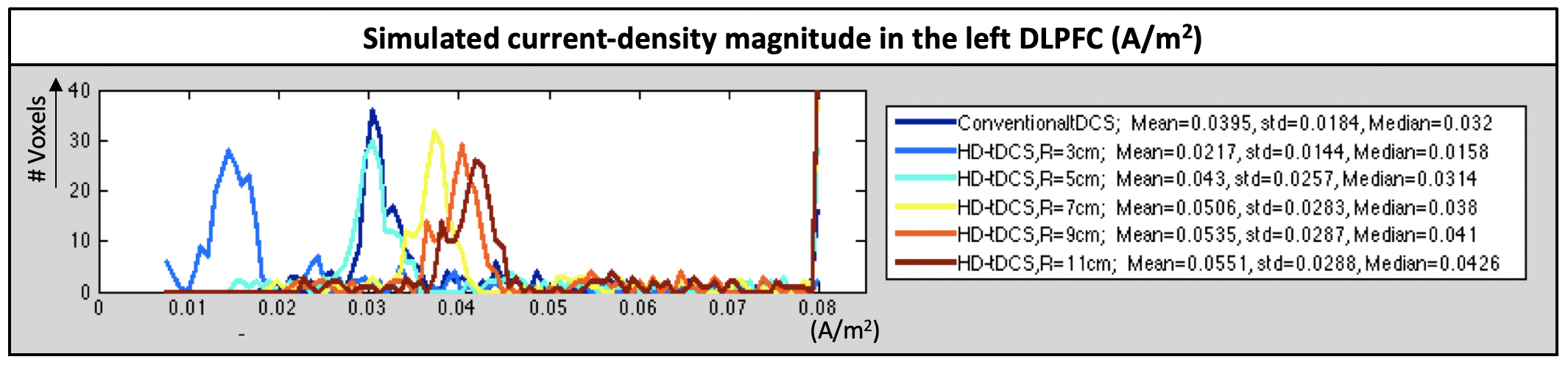
**

**Figure S14: Simulated current-density magnitudes for the Conventional montage, and 4x1 HD-tDCS montages with different separation distances (3, 5, 7, 9, 11 cm).** The histogram shows the current density magnitude in the left DLPFC ROI for different montages simulated. Here, note that the current density histogram for the 5cm HD montage (cyan) is similar to that of the conventional (dark blue).

**References:**

1. American Psychiatric Association. Diagnostic and statistical manual of mental disorders : DSM-5. American Psychiatric A, American Psychiatric Association DSMTF, editors. Arlington, VA: American Psychiatric Association; 2013.

2. Brainbox-Neuro. Brainsight Neuronavigation [Available from: <https://brainbox-neuro.com/catalogue/neuro-navigation/tms-navigation/brainsight-tms-navigation/>.

3. BrainProducts. EASYCAP GmbH [Available from: <https://www.brainproducts.com/productdetails.php?id=20>.

4. Jog M, et al. Concurrent Imaging of Markers of Current Flow and Neurophysiological Changes During tDCS. Front Neurosci. 2020;14:374.

5. Harms MP, et al. Extending the Human Connectome Project across ages: Imaging protocols for the Lifespan Development and Aging projects. NeuroImage. 2018;183:972-84.

6. Barnhill E, Kennedy P, Johnson CL, Mada M, Roberts N. Real-time 4D phase unwrapping applied to magnetic resonance elastography. Magn Reson Med. 2015;73(6):2321-31.

7. Friston KJ, Williams S, Howard R, Frackowiak RS, Turner R. Movement-related effects in fMRI time-series. Magn Reson Med. 1996;35(3):346-55.

8. Alsop DC, et al. Recommended implementation of arterial spin-labeled perfusion MRI for clinical applications: A consensus of the ISMRM perfusion study group and the European consortium for ASL in dementia. Magn Reson Med. 2015;73(1):102-16.

9. Goksu C, et al. Human in-vivo brain magnetic resonance current density imaging (MRCDI). NeuroImage. 2018;171:26-39.

10. Goksu C, Scheffler K, Siebner HR, Thielscher A, Hanson LG. The stray magnetic fields in Magnetic Resonance Current Density Imaging (MRCDI). Phys Med. 2019;59:142-50.

11. Yushkevich PA, et al. User-guided 3D active contour segmentation of anatomical structures: significantly improved efficiency and reliability. NeuroImage. 2006;31(3):1116-28.

12. Queval L. Biot Savart magnetic Toolbox 2020 [Available from: <https://www.github.com/lqueval/BSmag>.

13. Sallet J, et al. The organization of dorsal frontal cortex in humans and macaques. J Neurosci. 2013;33(30):12255-74.

14. Destrieux C, Fischl B, Dale A, Halgren E. Automatic parcellation of human cortical gyri and sulci using standard anatomical nomenclature. NeuroImage. 2010;53(1):1-15.

15. Rief W, et al. Assessing general side effects in clinical trials: reference data from the general population. Pharmacoepidemiol Drug Saf. 2011;20(4):405-15.

16. Thielscher A, Antunes A, Saturnino GB. Field modeling for transcranial magnetic stimulation: A useful tool to understand the physiological effects of TMS? Conference proceedings : Annual International Conference of the IEEE Engineering in Medicine and Biology Society IEEE Engineering in Medicine and Biology Society Annual Conference. 2015;2015:222-5.

17. Wagner S, et al. Investigation of tDCS volume conduction effects in a highly realistic head model. J Neural Eng. 2014;11(1):016002.

18. Opitz A, Paulus W, Will S, Antunes A, Thielscher A. Determinants of the electric field during transcranial direct current stimulation. NeuroImage. 2015;109:140-50.

19. Caparelli-Daquer EM, et al. A pilot study on effects of 4x1 high-definition tDCS on motor cortex excitability. Conference proceedings : Annual International Conference of the IEEE Engineering in Medicine and Biology Society IEEE Engineering in Medicine and Biology Society Annual Conference. 2012;2012:735-8.

20. Edwards D, et al. Physiological and modeling evidence for focal transcranial electrical brain stimulation in humans: a basis for high-definition tDCS. NeuroImage. 2013;74:266-75.
